# Evaluation of the *Trypanosoma brucei* 6-oxopurine salvage pathway as a potential target for drug discovery

**DOI:** 10.1371/journal.pntd.0006301

**Published:** 2018-02-26

**Authors:** Eva Doleželová, David Terán, Ondřej Gahura, Zuzana Kotrbová, Michaela Procházková, Dianne Keough, Petr Špaček, Dana Hocková, Luke Guddat, Alena Zíková

**Affiliations:** 1 Institute of Parasitology, Biology Centre, Czech Academy of Sciences, Branišovská, České Budějovice, Czech Republic; 2 The School of Chemistry and Molecular Biosciences, The University of Queensland, Brisbane, QLD, Australia; 3 Faculty of Science, University of South Bohemia, Branišovská, České Budějovice, Czech Republic; 4 The Institute of Organic Chemistry and Biochemistry of the Czech Academy of Sciences, Flemingovo nám. Prague, Czech Republic; KARI-Trypanosomiasis Res Centre, KENYA

## Abstract

Due to toxicity and compliance issues and the emergence of resistance to current medications new drugs for the treatment of Human African Trypanosomiasis are needed. A potential approach to developing novel anti-trypanosomal drugs is by inhibition of the 6-oxopurine salvage pathways which synthesise the nucleoside monophosphates required for DNA/RNA production. This is in view of the fact that trypanosomes lack the machinery for *de novo* synthesis of the purine ring. To provide validation for this approach as a drug target, we have RNAi silenced the three 6-oxopurine phosphoribosyltransferase (PRTase) isoforms in the infectious stage of *Trypanosoma brucei* demonstrating that the combined activity of these enzymes is critical for the parasites’ viability. Furthermore, we have determined crystal structures of two of these isoforms in complex with several acyclic nucleoside phosphonates (ANPs), a class of compound previously shown to inhibit 6-oxopurine PRTases from several species including *Plasmodium falciparum*. The most potent of these compounds have K_i_ values as low as 60 nM, and IC_50_ values in cell based assays as low as 4 μM. This data provides a solid platform for further investigations into the use of this pathway as a target for anti-trypanosomal drug discovery.

## Author summary

Human African Trypanosomiasis (HAT) is a life-threatening infectious disease caused by the protozoan parasite, *Trypanosoma brucei*. Current treatments suffer from low efficacy, toxicity issues and complex medication regimens. Moreover, an alarming number of these parasites are demonstrating resistance to current drugs. For these reasons, there is a renewed effort to develop new classes of modern therapeutics based upon the unique *T*. *brucei* cellular processes. One potential new drug target is 6-oxopurine phosphoribosyltransferase (PRTase), an enzyme central to the purine salvage pathway and whose activity is critical for the production of the nucleotides (GMP and IMP) required for DNA/RNA synthesis within this protozoan parasite. We demonstrated that *T*. *brucei* encodes two isoforms of hypoxanthine-guanine PRTases (HGPRT) and one hypoxanthine-guanine-xanthine PRTase (HGXPRT). The concurrent activity of these enzymes is required for the normal cell growth *in vitro*. Moreover, acyclic nucleoside phosphonates represent a promising class of potent and selective compounds as they inhibit the enzymes with K_i_ values in nanomolar range and exert cytotoxic effects on *T*. *brucei* cells grown *in vitro* with EC_50_ values in the single digit micromolar range. Our results provide a new foundation for further investigations of these compounds *in vivo* and suggest that 6-oxopurine salvage pathway represents a possible target for future drug discovery efforts directed at eliminating HAT.

## Introduction

*Trypanosoma brucei* is the etiological agent of Human African Trypanosomiasis (HAT) also known as “sleeping sickness”. HAT is a neglected disease that mainly affects Sub-Saharan countries, with ~70 million people at risk of infection [1–3]. The metacyclic trypomastigote form of *T*. *brucei* is primarily transmitted to humans by the bite of an infected tsetse fly. Once inside the mammalian host the parasite invades the bloodstream and lymph system. At this stage, the human host is mainly asymptomatic, a period that can last for months and up to years. However, when *T*. *brucei* crosses the blood-brain barrier, a degenerative neurological breakdown occurs characterized by continuous sleep-wake patterns. In the last stage, the human host falls into a coma and at this point the disease is fatal. A handful of drugs (pentamidine, eflornithine, nifurtimox, melarsoprol and suramin) is available to treat HAT at the different stages of the disease (e.g. haemo-lymphatic and brain infections). However, they are far from perfect drugs due to their low selectivity, high cost of production, high levels of toxicity, adverse side-effects and can have less than ideal routes of administration [4]. The increasing occurrence of resistance to these drugs is also of growing concern [5,6]. Therefore, new and more effective drugs that can be co-administered or replace the current treatments for this disease are urgently needed.

**Fig 1:**
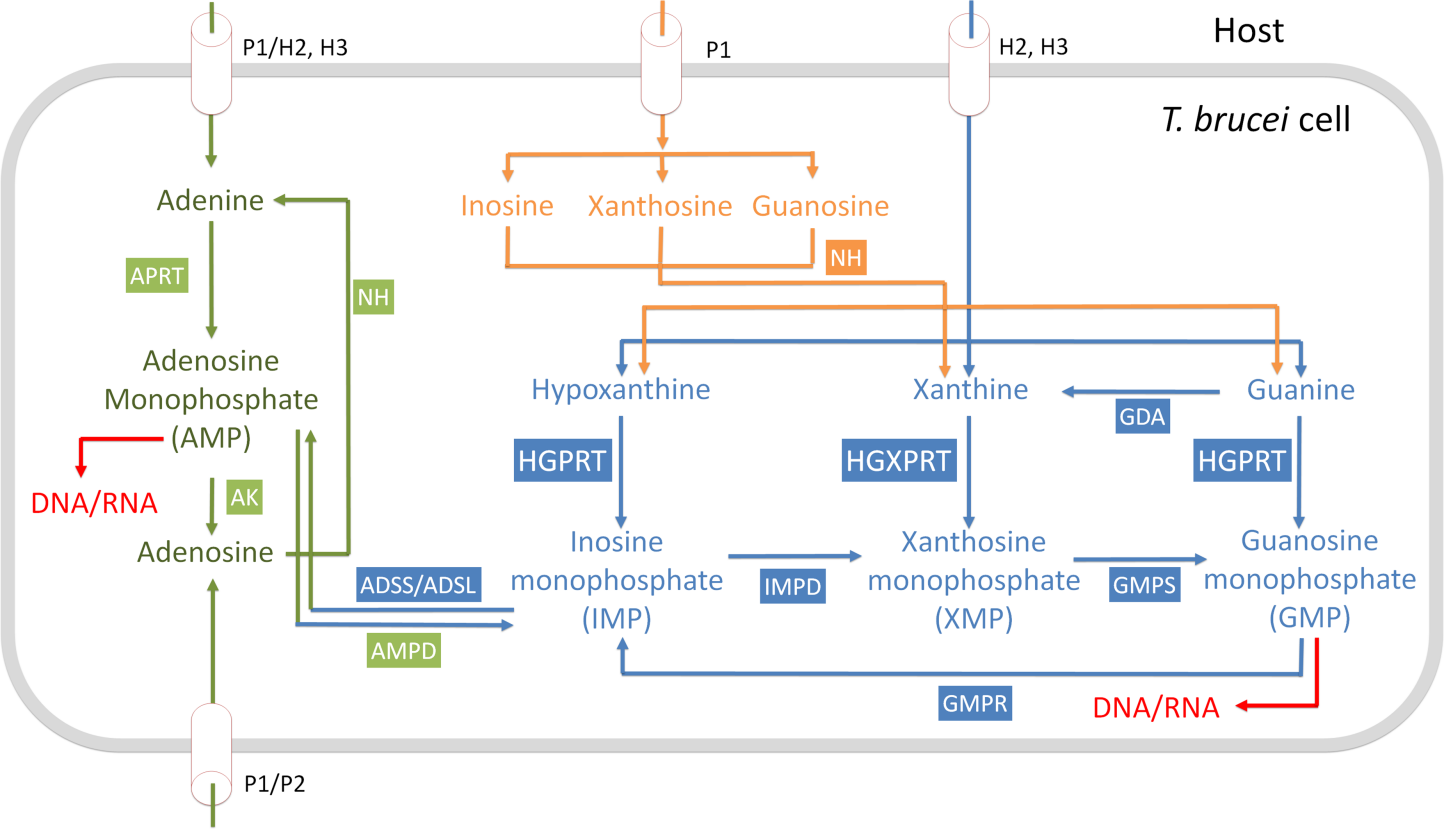
Purine salvage pathway of *Trypanosoma brucei* [17]. NH, nucleoside hydrolase; AK, adenosine kinase; APRT, adenine phosphoribosyl transferase; AMP, AMP deaminase; HGPRT, hypoxanthine guanine phosphoribosyl transferase; HGXPRT, hypoxanthine guanine xanthine phosphoribosyl transferase; IMPD, inosine-5´-monophopshate dehydrogenase; GMPS, GMP synthase; GDA, guanine deaminase; GMPR, GMP reductase; AMP DA, AMP deaminase; ADSS, adenylosuccinate synthetase; ADSL, adenylosuccinate lyase.

**Fig 2:**
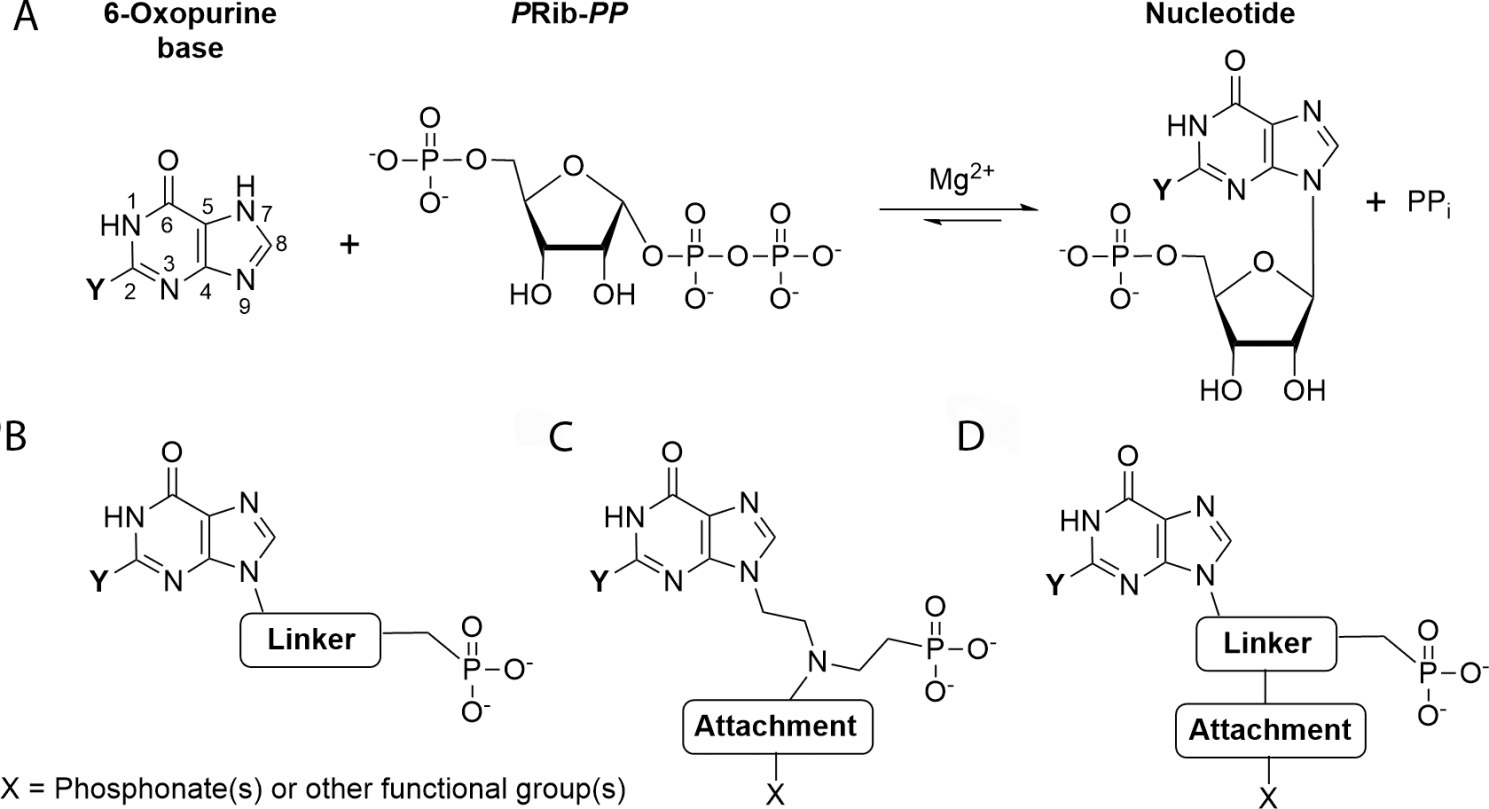
(A) Reaction catalyzed by the 6-oxopurine PRTases. (B–D) General structures of ANPs. Single chain ANPs (B); aza-ANPs (C); and branched ANPs with an attachment at one of the first two carbons from the N^9^ nitrogen in the base (D). When xanthine, guanine and hypoxanthine is the base, Y = OH, Y = NH_2_ and Y = H, respectively.

The complete sequencing of the *T*. *brucei* genome has identified some differences in metabolism between the parasite and the human host, which could lead to the discovery of new drug treatments [7,8]. One significant difference between the human host and this parasite is in the respective enzymes they have available for the synthesis of the nucleoside monophosphates required for the production of their DNA and RNA. In *T*. *brucei* there is a complete reliance on the purine salvage pathways, obtaining the purine bases from the host, whereas in humans both the *de novo* pathway and the salvage pathways are present [9–12]. The trypanosome purine salvage pathway is comprised of several salvage enzymes (i.e. nucleoside hydrolases, 6-oxopurine PRTases, adenine PRTase, adenosine kinase) and interconversion enzymes (i.e. AMP deaminase, adenylosuccinate lyase (ADSL), adenylosuccinate synthetase (ADSS), guanine deaminase, GMP synthase (GMPS), GMP reductase and inosine-5´-monophopshate dehydrogenase) (Fig 1). Importantly, there are constitutive differences between humans and *T*. *brucei* within the salvage pathways themselves. For example, *T*. *brucei* has three 6-oxopurine PRTase isoforms whereas there is only one 6-oxopurine PRTase in humans. These enzymes catalyze the transfer of the ribose 5'-phosphate moiety from 5-phospho-α-D-ribosyl-1-pyrophosphate (*P*Rib-*PP*) to their respective purine bases (hypoxanthine, guanine or xanthine) (Fig 2A). Thus, due to the central importance of these proteins in RNA/DNA synthesis, inhibition of these enzymes is suggested to be a viable approach to antiprotozoal drug therapy [13–16].

Acyclic nucleoside phosphonates (ANPs) are a family of antiviral compounds that have been shown to also inhibit plasmodial and mycobacterial 6-oxopurine PRTases [18–20]. The basic structure of these compounds consists of a nucleobase connected to a phosphonate group by a variety of chemical linkers. In some ANPs, this linker consists solely of carbon atoms while others have oxygen or nitrogen atom(s) to replace the carbon atoms [16,21–23] (Fig 2B). More elaborate ANPs have a functional attachment at the position one, two or three atoms along the linker (Fig 2C and 2D). A crucial property of the ANPs is the presence of a stable CH_2_-P bond in the phosphonate moiety [19,24] (Fig 2B, 2C and 2D). This means such compounds are not susceptible to hydrolysis within the cell of the host or pathogen [19,21,22,25–28]. However, the ANPs possess negative charges on the phosphonic oxygen atoms, restricting cell permeability. In the case of the bisphosphonates (Fig 2C and 2D), transport across the cell membranes is even more problematic [29]. To overcome this issue, the cell permeability was enhanced by attaching lipophilic or hydrophobic groups to the phosphorus atom either by a phosphoramidate or ester bond. Once inside the cell, these groups are hydrolyzed to produce the active parent compound [29–31].

Here, we show that two of the three *T*. *brucei* isoforms of the 6-oxopurine PRTases are identified as HGPRTs as a reflection of their substrate preference for hypoxanthine and guanine and the third isoform is identified as HGXPRT reflecting that it uses all three naturally occurring bases. We have performed double RNAi silencing of the HGPRTs and HGXPRT to show that the 6-oxopurine PRTases are crucial for the survival of *T*. *brucei in vitro*. We have also determined the crystal structures of two of the 6-oxopurine PRTase isoforms in complex with several ANPs and showed that prodrugs of these inhibitors possess antitrypanosomal activity in cell based assays.

## Material and methods

### *Trypanosoma* culture and cell lines

The *T*. *b*. *brucei* Lister 427 procyclic form (PF) was cultivated in SDM-79 medium containing 10% FBS at 27°C [32]. The bloodstream form (BF) *T*. *b*. *brucei* Lister 427 and single marker (SM) strains were cultivated in HMI-9 medium and 10% FBS at 37°C in a humidified atmosphere at 5% CO_2_ [32]. The HMI-9 medium contains hypoxanthine at 1 mM concentration, while other purines are present in negligible amounts [33]. The SM cell line constitutively expresses the ectopic T7 RNA polymerase and tetracycline repressor and was used for the tetracycline inducible expression of dsRNA and the v5-tagged proteins.

The 431 bp and 481 bp fragments of *hgprt-I* (Tb927.10.1400) and *hgxprt* (Tb927.10.1390) open reading frames, respectively, were PCR amplified from *T*. *brucei* BF427 genomic DNA with the oligonucleotides listed in S1 Supplementary File and cloned into the p2T7-177 plasmid [34] utilizing BamHI, HindIII or XhoI restriction sites inherent in the primers.

For the inducible expression of v5-tagged HGPRT-I, HGPRT-II and HGXPRT, the coding sequences of the respective genes were PCR amplified using oligonucleotides listed in S1 Supplementary File. The obtained PCR fragments were cloned into the pT7_N-term-v5 vector derived from pT7 vector [35] using BamHI and XbaI restriction enzymes in order to maintain the open reading frames of the N-terminal 3 x v5 tag and the respective genes.

The p2T7-177 and pT7_N-term-v5 plasmids containing fragments or open reading frames, respectively, of *hgprt-I*, *hgprt-II* and *hgxprt* genes were linearized with NotI enzyme and transfected into the BF SM cell line as described previously. The induction of dsRNA and v5-tagged protein expression was triggered by the addition of 1 μg/ml of tetracycline into the medium. Cell densities were measured using the Z2 Cell Counter (Beckman Coulter Inc.). Throughout the analyses, BF cells were maintained in the exponential mid-log growth phase (between 1x10^5^ to 1x10^6^ cells/ml).

### Immunofluorescence assay

For immunofluorescence 1 × 10^8^ of BF trypanosomes were washed with PBS supplemented with 10 g/l glucose, and spread onto slides coated with polylysine (100 μg/ml; Sigma). The cells were fixed with 3.7% formaldehyde in phosphate-buffered saline (PBS), washed with PBS, and permeabilized with 0.1% triton X-100 in PBS. The cells were then washed with 1xPBS-T (0.05% Tween). After blocking with 5.5% FBS, the respective slides were incubated for 1 hr in PBS-T plus 3% BSA with the following primary antibodies: monoclonal anti-v5 (1:200, Invitrogen), anti-enolase (1:400, [36]) and anti-hexokinase (1:400, a gift from Paul Michels). After washes, the slides were incubated in the dark for 1 hr with the following secondary antibodies: goat anti-rabbit IgG (H + L) Texas Red conjugate (1:400, Life Technologies) or goat anti-mouse IgG (H + L) FITC conjugate (1:400, Sigma). The slides were washed and mounted in Vectashield containing 1.5 μg/ml DAPI (Life Technologies).

### SDS-PAGE and Western blot

Protein samples were separated on SDS-PAGE, blotted onto a PVDF membrane (PALL) and probed with the appropriate monoclonal (mAb) or polyclonal (pAb) antibodies. This was followed by incubation with a secondary HRP-conjugated anti-rabbit or anti-mouse antibody (1:2000, BioRad). Proteins were visualized using the Clarity Western ECl Substrate (Biorad) on a ChemiDoc instrument (BioRad). ImageLab software version 4.1 (Bio-Rad) was used for image acquisition and densitometric analysis of the blots. The PageRuler prestained protein standard (ThermoFisher Scientific) was used to determine the size of detected bands. Primary antibodies used in this study were: mAb anti-v5 epitope tag (1:2000, ThermoFisher Scientific), mAb anti-mtHsp70 (1:2000, [37]), pAb anti-enolase (1:1000), pAb anti-hexokinase (1:1000), pAb anti- HGPRT-I (1:100, this work) and pAb anti-HGXPRT (1:1000, this work).

### Digitonin subcellular fractionation

Whole cell lysates (WCLs) were prepared from *T*. *brucei* PF427 and BF427 strains. SoTe/digitonin fractionation was performed to obtain cytosolic and organellar fractions. Briefly, 1x10^8^ cells were harvested by centrifugation, washed in PBS-G, resuspended in 500 μl SoTE (0.6 M Sorbitol, 2 mM EDTA, 20 mM Tris-HCl pH 7.5) and lysed with 500 μl SoTE containing 0.03% digitonin. The cells were then incubated on ice for five minutes followed by centrifugation (4°C, 4500 g, 3 min). WCL, supernatant representing the cytosolic fraction (CYTO) and pellet representing the organellar membrane fraction (ORG) were fractionated by SDS-PAGE and analysed by immunoblotting.

### AlamarBlue assay

An Alamar Blue Assay was performed in a 96-well plate format to screen for sensitivity of *T*. *brucei* BF427 strain to ANPs. Parasites at 5×10^3^ cells/ml were incubated with different drug concentrations (two-fold serial dilutions) at 200 μl final volume of HMI-9 with 10% FBS / well for 48 hours at 37°C and 5% CO_2_. Afterward, Alamar Blue reagent (20 μl) was added to each well and cells were incubated for an additional 12 hours under the same conditions. The fluorescence signal was quantified using a Tecan Infinite M200 plate reader (Excitation 560 nm–Emission 590 nm). The EC_50_ values were calculated using non-linear regression in GraphPad Prism5.

### Immunoprecipitation

5 × 10^8^ cells were washed in PBS and lysed in immunoprecipitation (IP150) buffer (Tris 10 mM pH 7.2, 10 mM MgCl_2_, KCl 150 mM, 1% Triton, and protease inhibitor cocktail (Roche Applied Science) for 1 hour on ice. The v5-tagged HGPRT-I, -II and HGXPRT complexes were purified using magnetic beads (Dynabeads M-280, sheep anti-mouse IgG, ThermoFisher Scientific) charged with monoclonal anti-v5 antibody. The lysate was incubated with the charged beads for 2 h (4°C). The beads were then washed three times in IP150 buffer before the bound protein complexes were released by the addition of SDS-PAGE loading buffer and boiling (97°C, 10 min).

### Protein crosslinking

Purified HGPRT-I, HGPRT-II and HGXPRT were dialyzed to 20 mM HEPES, 1 mM EDTA, at pH 8.0 and diluted to 0.1 mg/ml. A non-crosslinked sample was set aside and dimethylsuberimidate (DMS) was added from fresh 20 mg/ml stock solution in water to give a final concentration of 1 mg/ml. Crosslinking was stopped at different time points by mixing samples with SDS-PAGE loading buffer. The crosslinked proteins were resolved by SDS-PAGE on 15% gels and stained by Coomassie Brilliant Blue dye.

### Synthesis of the inhibitors and their prodrugs

The inhibitors and their prodrugs described here were synthesized as described previously [21,23,28,29].

### Expression and purification of HGPRT-I and HGXPRT for in vitro activity assays

The *T*. *brucei hgprt-I*, *hgprt-II and hgxprt* genes were PCR amplified using oligonucleotides listed in S1 Supplementary File, digested with BamHI and XhoI enzymes and ligated into the pSKB3 expression vector. The pSKB3 vector carries a 6x His-tag and an AcTEV cleavage site upstream of BamHI restriction site. A BamHI restriction site was introduced at the 5' end of the respective genes instead of the initiation methionine codon to maintain the 6x His-tag and AcTEV recognition motif in frame with the genes. The verified plasmids were transformed into the One Shot BL21(DE3) Chemically Competent *E*. *coli* cells (Life Technologies). The bacterial cells were grown in LB medium until the OD_600_ reached ~ 0.4. The expression of the recombinant proteins was induced by the addition of 1 mM isopropyl β-D-1-thiogalactopyranoside (IPTG). After three hours of induction the cells were harvested, washed in PBS and resuspended in the STE buffer (50 mM Tris-HCl pH 8.0, 150 mM NaCl, 1 mM EDTA) supplemented with the cOmplete EDTA-free protease inhibitor cocktail (Roche, Switzerland). Cells were lysed by 1% Triton X-100 and lysozyme (1 mg/ml) for 30 min at 4°C, followed by DNase I treatment (5 μg/ml, 30 min rotation at 4°C) in the presence of MgCl_2_ (30 mM). The lysates were then centrifuged and filtered before loading onto the Ni-NTA column (1ml Histrap HP, GE Healthcare). The 6xhis-tagged recombinant proteins were purified under native conditions using the ÄKTA prime plus instrument accordingly to the product manual. Elution fractions were analyzed by SDS-PAGE, and those containing HGPRT-I, -II or HGXPRT were pooled and dialyzed against 50 mM Tris-HCl, pH 8.0, 150 mM NaCl, 10% glycerol. Protein concentrations were determined using Bradford protein assay (BioRad) with BSA as standards. The HGPRT-I and HGXPRT recombinant proteins were sent for antibody production to Davids Biotechnology (Regensburg, Germany). Recombinant HGPRT-I, HGPRT-II and HGXPRT were subsequently used in all *in vitro* assays.

### Enzyme activity and determination of kinetic constants

The activity of HGPRT-I, HGPRT-II and HGXPRT was measured by a continuous spectrophotometric assay measuring the conversion of hypoxanthine, guanine and xanthine to IMP, GMP and XMP, at 245 nm, 257.5 nm and 255 nm, respectively [38]. The concentration of enzyme in the assay (varied from 75–200 μg/ml for the two enzymes) and the concentration of *P*Rib-*PP* was kept at 1 mM. The reaction was carried out using a 1.0 cm path length with a UV Visible spectrometer 1601 (Shimadzu) in the reaction buffer (0.1 M Tris, pH 8.4; 0.11 M MgCl_2_; at 25°C). The initial velocity (V_0_ [μM/min]) was calculated using Beer-Lambert law for different concentration of the purine substrate molecules: A = Δε*L*c where A is measured absorbance, L is length of cuvette, Δε is constant (5817 M^-1^*cm^-1^ for guanine, 2283 M^-1^*cm^-1^ for hypoxanthine and 4685 M^-1^*cm^-1^ for xanthine [39] and c is concentration of the product formed in one minute (V_0_). The K_m_, V_max_ and k_cat_ values were calculated using GraphPad Prism5 according to Michaelis-Menten kinetics.

### Inhibition studies

The K_i_ values for HGPRT-I, HGPRT-II and HGXPRT were determined in buffer containing 0.1 M Tris, pH 8.4; 0.11 M MgCl_2_; at 25°C. The concentrations of inhibitors in the assay ranged from 1 nM to 100 μM, depending on the steady-state kinetics of the respective enzyme at that concentration of inhibitor. The reaction was preceded by 1 minute incubation of the respective enzyme with the given inhibitor. The K_i_ values were calculated using non-linear regression in GraphPad Prism5.

### Expression and purification of HGPRT-I and HGXPRT for crystallographic studies

Expression and purification of HGPRT-I was performed following a procedure described previously [16]. Expression and purification of HGXPRT was carried out as above but with some minor modifications. Induction of expression was at 37°C for 3 hours upon the addition of 0.5 M IPTG. Cells containing the expressed protein were centrifuged at 4°C at 4000 x g for 15 minutes. The cell pellet was washed with 1xPBS and centrifuged a second time at 4°C at 4000 x g for 10 minutes. It was then stored at -80°C until purification. The cell pellet was then re-suspended in 50 mM Tris-HCl, 150 mM NaCl, 1 mM EDTA, 1% Triton X-100, pH 8 in the ratio of 39 mL of buffer to 1 g of pellet. Cells were lysed by the addition of lysozyme (1 mg per ml of cells) in the presence of protease inhibitor cocktail (Roche) (1 tablet per 50 mL of cells) and incubated on ice for 30 min. The cells were then sonicated for four cycles for 30 seconds at 20% power with 1 minute between cycles. 1 M MgCl_2_ and DNase at 50 μg/ml of cells were then added prior to incubation at 4°C for 10 minutes. The soluble fraction was separated by centrifugation at 4°C at 10000 x g. The recombinant enzyme was purified using a Ni^2+^ chromatography column (Profinity IMAC resin) at 4°C. The elution buffer was 50 mM Tris-HCl, 500 mM NaCl, 30 mM imidazole, 10% glycerol, pH 8. The eluent was then dialyzed into 50 mM Tris-HCl, 12 mM MgCl_2_, 10% glycerol, pH 8 at 4°C overnight. For crystallographic studies, the enzyme was concentrated to 10 mg/mL using an Amicon concentrator (model 12–76 psi) and an Amicon Ultra-15 centrifugal device. All samples were subsequently stored at −80°C. Sample purity was assessed by (12%) SDS-PAGE. A Direct Detect spectrometer used to determine protein concentration. This method measures the absorbance due to the presence of amide bonds in the polypeptide at A_220_.

### Crystallization and structure determination of HGXPRT and HGPRT

Initial screening of crystallization conditions was performed by the hanging drop vapor-diffusion method using PACT *premier*, INDEX HT (Hampton research), Proplex, PEG ion crystallization screens providing the lead crystals for optimization. Crystals for diffraction studies were obtained by incubating HGPRT-I or HGXPRT with the different ANPs on ice for 15 min. 1 μL of the HGPRT-I.ANP complex or HGXPRT.ANP complex was then mixed with 1 μL of well solution. For HGPRT-I the enzyme concentration was 28 mg/ml and the inhibitor concentration was 1.3 mM. The reservoir solution was 25% PEG 3350, 0.2 M lithium sulfate and 0.1 M Bis-Tris with the pH between 5 and 7. For HGXPRT the enzyme concentration was 5 mg/ml and the inhibitor concentration was 1.3 mM. The reservoir solution was 25% PEG 3350, 0.2 M lithium sulfate and 0.1 M Bis-Tris with the pH 5. Prior to cryocooling, all crystals were transferred to a cryoprotectant solution containing the reservoir, 20% glycerol and 2 mM of each ligand. Crystals were subsequently transported to the Australian Synchrotron and robotically placed in the cryostream (100 K) on beamline MX2 for HGPRT-I.ANPs complexes and MX1 for HGXPRT.ANPs complexes. X-ray data were collected remotely by BLU-ICE [40]. Data were, integrated, scaled and merged using XDS [41]. The structure of *T*. *brucei* HGPRT-I in complex with single chain ANP (PDB code 5JSQ) was used as starting model for molecular replacement for HGPRT. The structure of *Trypanosoma cruzi* HGPRT in complex with *P*Rib-*PP* and HPP **(**PDB code 1TC2) was used as starting model for molecular replacement for HGXPRT, which was then phased using the program PHASER [42]. Subsequent refinement and model building was performed with PHENIX 1.7.3 [43] and COOT 0.8.2 [44].

### Accession numbers

The coordinates and structure factors for all six complexes have been deposited in the protein data bank. The access codes for HGPRT.6, HGPRT.7, HGPRT.1 HGPRT.2, HGXPRT.7, HGXPRT.2 are 6APS, 6APT, 6APU, 6APV, 6AQO and 6AR9, respectively.

## Results

### Steady state kinetic analysis of HGPRT-I, HGPRT-II and HGXPRT

*T*. *brucei* genome [7] is annotated to contain three putative HGPRT genes: Tb927.10.1400 (HGPRT-I), Tb927.10.1470 (HGPRT-II) and Tb927.10.1390 (HGPRT-III). The genes for *hgprt-I* and *hgprt-II* are almost identical sharing 98% identity at the nucleotide level. At the amino acid level the HGPRT-I and HGPRT-II proteins differ at amino acid positions 49, 54, 206, 207 and 210. The last three amino acids are located at the C-terminus. Hence, HGPRT-I ends with the GEAKR sequence while HGPRT-II ends with VKAKL. The tripeptide, AKL, follows the highly conserved consensus sequence S/A-K/R-L/M for peroxisomal targeting and indeed this protein is predicted to be localized in peroxisomes by the PTS1 Predictor programme. The gene for *hgprt-III* differs significantly from the *hgprt-I* and *-II* isoforms sharing only 56% identity at the nucleotide level and 35% identity at the amino acid level. The product of this gene uses all three naturally occurring 6-oxopurines (*i*.*e*. guanine, hypoxanthine and xanthine, see below). Therefore, we identify this isoform as HGXPRT.

To explore the specificity of HGPRT-I, HGPRT-II and HGXPRT steady-state kinetic constants were determined for the three naturally occurring bases (Table 1). HGXPRT catalyzes the synthesis of GMP, IMP and XMP with k_cat_ values of 1.36, 2.4 and 3.66 s^-1^, respectively. These rates are comparable with those for *Leishmania donovani* HGPRT and XPRT [45] and *P*. *falciparum* HGXPRT [38]. HGPRT-I and HGPRT-II catalyze the conversion of guanine and hypoxanthine at much slower rate (Table 1) and show no activity for xanthine at the concentrations tested confirming the annotation of these enzymes as HGPRTs. Considering that HGPRT-I and HGPRT-II are almost identical at the amino acid level, the K_m_ and k_cat_ values differ significantly for the tested purine nucleobases (Table 1). Apart from the three residues in the peroxisome signalling sequence, these two enzymes also differ at amino acid position 49 and 54, the latter one being in the middle of the PP_i_ binding pocket.

**Table 1:** Kinetic constants of the naturally occurring 6-oxopurine bases for HGPRT-I, HGPRT-II and HGXPRT.

Substrate	K_m_ (μM)	k_cat_ (s^-1^)	k_cat_/ K_m_ (μM^-1^s^-1^)
**HGPRT-I**			
Guanine	15.9 ± 4.8	0.38	0.02
Hypoxanthine	13.7 ± 2.9	0.26	0.18
Xanthine	No activity detected		
**HGPRT-II**			
Guanine	7.1±1.8	0.05	0.007
Hypoxanthine	4.2 ± 0.9	0.03	0.007
Xanthine	No activity detected		
**HGXPRT**			
Guanine	32 ± 4.8	1.36	0.04
Hypoxanthine	30.6 ± 7.8	2.4	0.08
Xanthine	31.9 ± 7.5	3.66	0.11

In summary, this data shows that all three *T*. *brucei* 6-oxopurine PRTases are able to bind hypoxanthine and guanine bases and that HGXPRT is a promiscuous enzyme possessing affinity for all three naturally occurring substrates with no strong preference for either one.

### Subcellular localization of HGPRT-I, -II and HGXPRT in BF *T*. *brucei*

**Fig 3:**
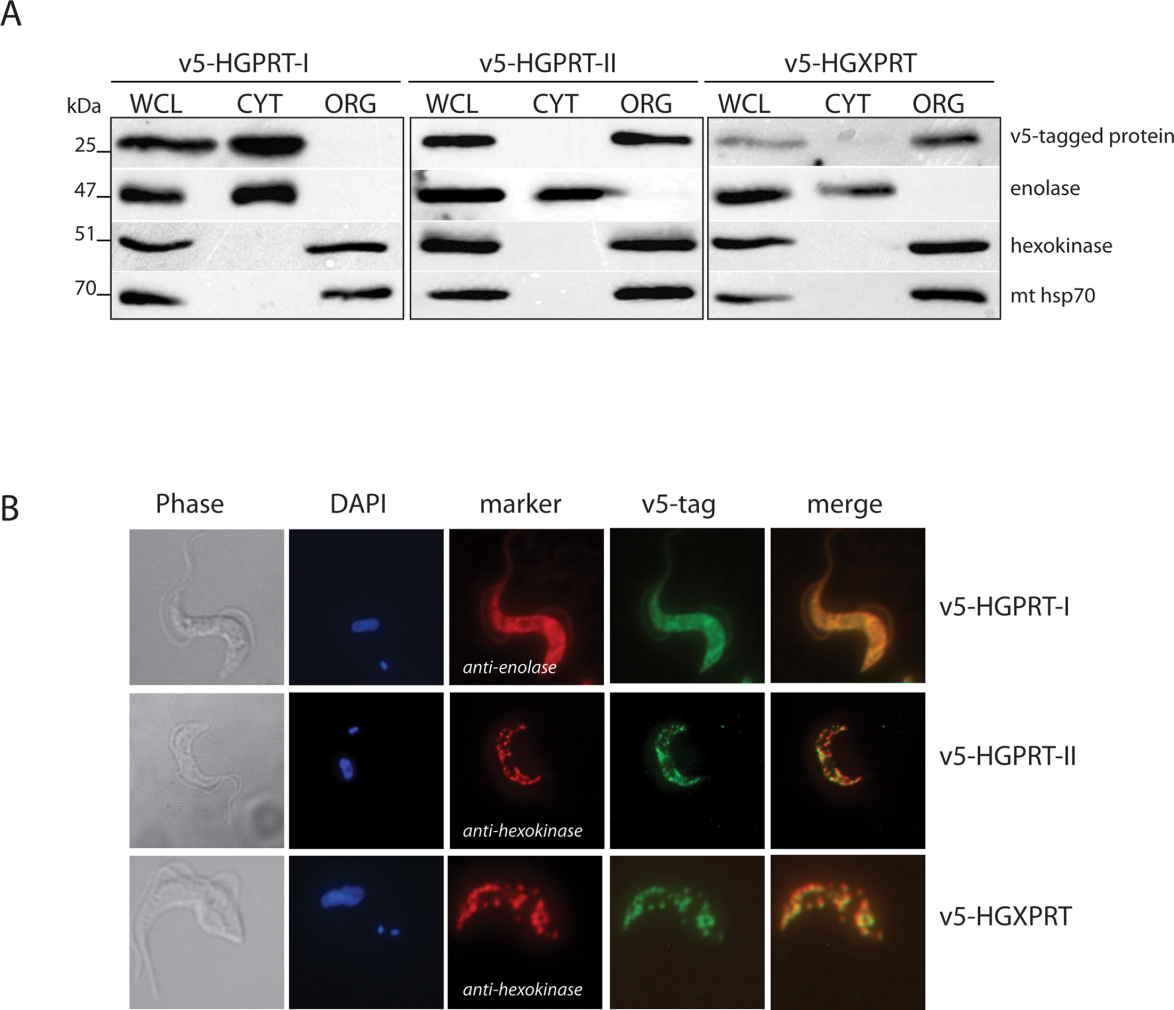
Subcellular localization of HGPRT-I, HGPRT-II and HGXPRT in the bloodstream form of *T*. *brucei*. (A) Immunoblot analysis of BF cells over-expressing v5-tagged HGPRT-I, HGPRT-II and HGXPRT was performed to reveal the subcellullar localization of these proteins. Cytosolic (CYT) and organellar (ORG) fractions were obtained by digitonin fractionation. Purified fractions were analyzed by immunoblot with the following antibodies: anti-V5, anti-enolase (cytosol), anti-hexokinase (organellar fraction, glycosomes), anti-mt hsp70 (organellar fraction, mitochondrion). The relevant sizes of the proteins are indicated on the left. (B) Immunofluorescence microscopy of the same cell lines as in (A) was used to determine subcellular localization of the v5-tagged HGPRT-I, HGPRT-II and HGXPRT proteins within the cell. HGPRT-I, HGPRT-II and HGXPRT were visualized by immunostaining using a monoclonal anti-v5 antibody and anti-mouse secondary antibody conjugated with fluorescein isothiocyanate (FITC). Antibodies against enolase and hexokinase served to mark cytosolic and glycosomal localization, respectively. The DNA content (nucleus and kinetoplast) was visualized using DAPI (4,6-diamidino-2-phenylindole).

The surprising promiscuity of HGXPRT *in vitro* prompted us to investigate the properties of the *T*. *brucei* 6-oxopurine PRTases *in vivo*. To decipher the subcellular localization of HGPRT-I, -II and HGXPRT, the respective genes were N-terminally tagged with a v5 tag and their expression was triggered by adding tetracycline into the medium. Differential cell lysis was performed using digitonin, which at low concentrations, only dissolves the plasma membrane. The subsequent Western analyses of the cytosolic (CYT) and organellar (ORG) fractions established the purity of the extracted fractions. The cytosolic enolase, the mitochondrial hsp70 and glycosomal hexokinase were confined within their respective subcellular fractions and served as suitable controls. Notably, the v5-tagged HGPRT-II and HGXPRT were detected in the organellar fraction while HGPRT-I was present in the cytosolic fraction (Fig 3A). To determine if HGPRT-II and HGXPRT are localized to the mitochondrion or in the glycosome, the cells expressing the v5-tagged proteins were fixed and the respective enzymes were detected by immunostaining using an anti-v5 antibody (Fig 3B). Direct immunofluorescence showed that HGPRT-II and HGXPRT co-localize with hexokinase, indicating that these isoforms are in the glycosome. This is in agreement with the presence of the PTS1 tripeptide motif at the C-terminal end of these two proteins. Localization of HGPRT-I was confirmed to be cytosolic as the v5 signal co-localized with the cytosolic marker, enolase (Fig 3B).

### Differential expression of HGPRT-II in the PF and BF of *T*. *brucei*

**Fig 4:**
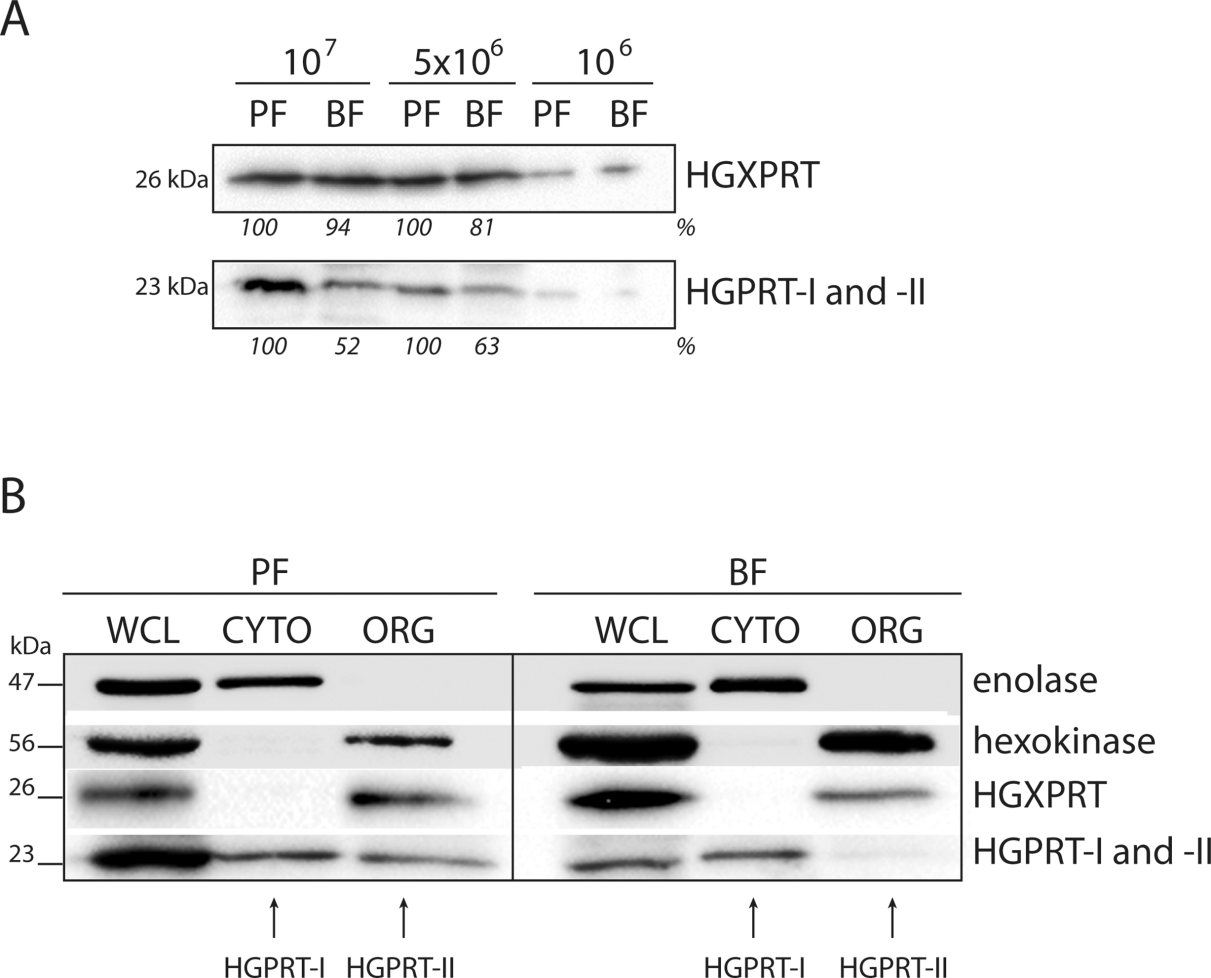
Expression of HGPRT-I, HGPRT-II and HGXPRT in the PF and BF cells. (A) The steady state abundance of HGPRT-I, HGPRT-II and HGXPRT was determined in PF and BF cells by Western blot analysis of whole cell lysates. Densitometric analysis was performed using the Image Lab 4.1 software and the number beneath the blots represents the abundance of immunodetected proteins expressed as a percentage of the PF sample. (B) Cellular fractionation of PF and BF cells was used to distinguish between the localization of cytosolic HGPRT-I and glycosomal HGPRT-II. Antibodies against hexokinase and enolase were used to mark the cytosol and glycosome, respectively. The protein marker sizes are indicated on the left.

Since the purine availability differs between the culture media used to grow PF and BF *T*. *brucei* cells, we examined the expression levels of the three different isoforms of 6-oxopurine PRTases in the two life stages. Whole cells lysates from 10^7^, 5x10^6^ and 10^6^ cells were fractionated on SDS-PAGE. The steady-state abundance of HGPRT-I, -II and HGXPRT was detected using Western blot analysis with polyclonal antiserums against HGXPRT and HGPRT-I. The antiserum raised against HGPRT-I also cross-reacts with HGPRT-II as the amino acid sequences of these two isoforms are virtually identical. While expression of HGXPRT appears to be unaffected in the two life cycle stages, the steady state abundance of HGPRT-I and–II was significantly decreased (approximately by 40–50%) in BF cells (Fig 4A). To distinguish between HGPRT-I and HGPRT-II isoforms, the 1x10^8^ PF and BF cells were lysed using a low concentration of digitonin allowing the cytosolic and organellar fractions to be examined using the anti-HGPRT-I serum (Fig 4B). The cytosolic signal for HGPRT-I remained similar between the PF and BF samples, but the signal for the glycosomal HGPRT-II was not detectable in the BF sample suggesting that expression of this protein is down-regulated in the infectious stage of the parasite.

### HGPRT-I and HGXPRT are essential for growth of the BF *T*. *brucei* cells

**Fig 5:**
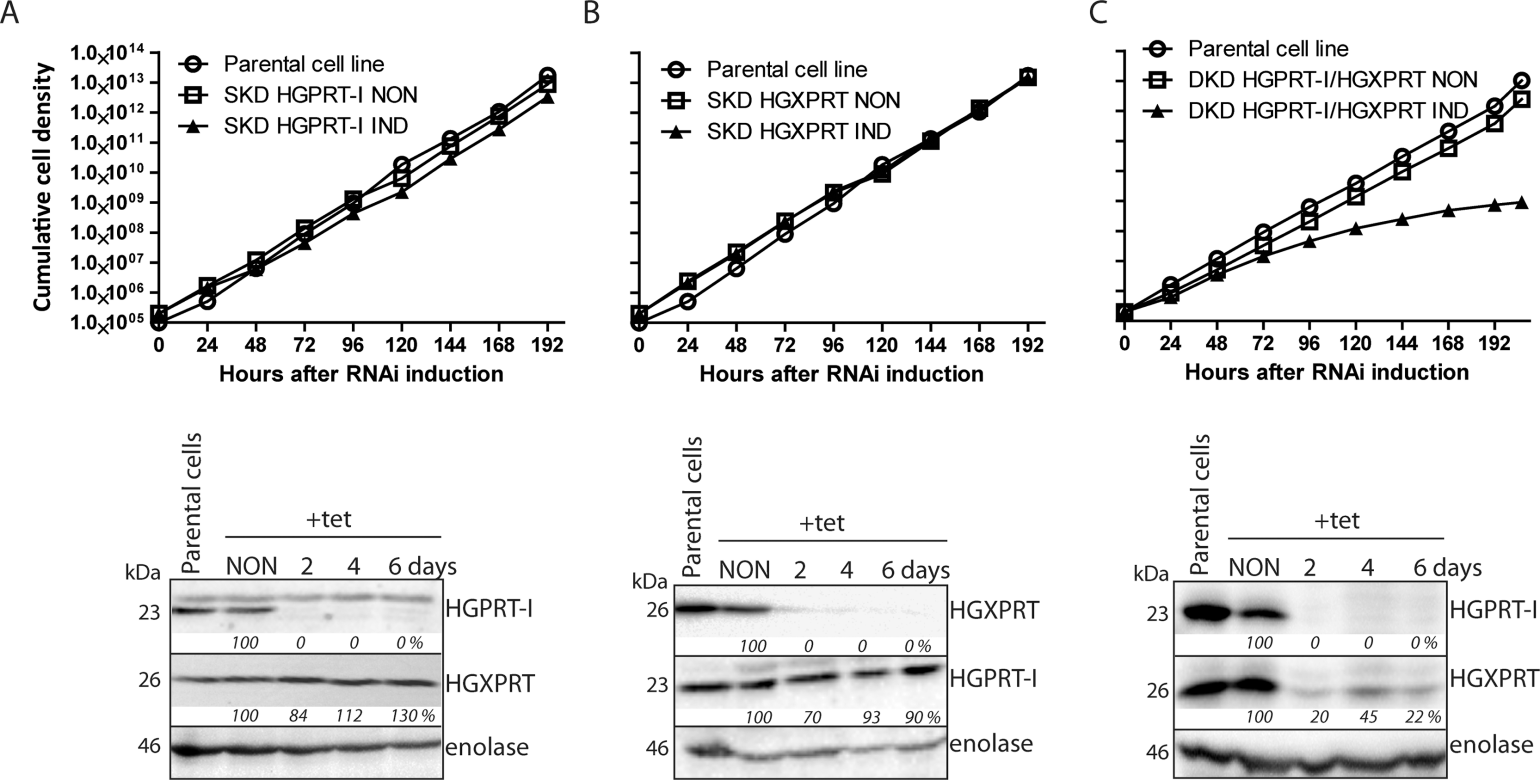
Effects of RNAi silencing of HGPRT-I, HGXPRT and simultaneous RNAi silencing of HGPRT-I/HGXPRT on *T*. *brucei* BF cell growth. Growth curves of the noninduced (NON) and RNAi induced (IND) cells in which the expression of HGPRT-I (A) HGXPRT (B) and HGPRT-I/HGXPRT (C) was RNAi silenced. Cells were cultured in HMI-9^full^ media and the cumulative cell number was calculated from cell densities adjusted by the dilution factor needed to see the cultures at 10^5^ cells/ml each day. The figure is representative of at least three independent RNAi inductions. The steady-state abundance of HGPRT-I and HGXPRT in noninduced (NON) RNAi cells and in cells induced with tetracycline for 2, 4 and 6 days (A, B and C, bottom panels) was determined by immunoblotting using specific anti-HGPRT-I and anti-HGXPRT serums. Densitometric analysis was performed using the Image Lab 4.1 software and the determined values were normalized to an enolase loading control signal. The protein marker sizes are indicated on the left.

In order to investigate if the activity of HGPRT-I and HGXPRT is essential for *in vitro* growth of BF cells we generated three RNAi cell lines: single knock-down (SKD) of HGPRT-I, SKD of HGXPRT and double knock-down (DKD) of both HGPRT-I and HGXPRT. It should be noted that RNAi against HGPRT-I also silences the residual expression of HGPRT-II in BF cells as the DNA fragment used for dsRNA expression is identical for these two isoforms. However since the HGPRT-II expression is undetectable in BF cells (Fig 3B) we kept the simplified labelling SKD HGPRT-I and DKD HGPRT-I/HGXPRT. No significant growth phenotype was observed for SKD HGPRT-I and SKD HGXPRT cell lines in HMI-9 medium containing 1 mM hypoxanthine (Fig 5A and 5B). The efficiency and specificity of RNAi was verified by Western blot using anti-HGPRT-I and anti-HGXPRT serums. Upon RNAi HGPRT-I silencing virtually none of this protein remained after two days, while the levels of HGXPRT remained largely unaffected (Fig 5A, bottom panel). In the flipped experiment, where HGXPRT was silenced by RNAi induction no signal for this enzyme was detected by the second day, while the levels of HGPRT-I remained unchanged (Fig 5B, bottom panel). These results show that HGPRT-I and HGXPRT possess interchangeable roles in hypoxanthine salvage and they are in full agreement with the enzyme kinetics that show both HGPRT-I and HGXPRT are able to efficiently convert hypoxanthine to IMP. When both, HGPRT-I and HGXPRT were silenced simultaneously, a significant growth phenotype appeared 72 hours after the tetracycline addition (Fig 5C). Western blot analysis revealed elimination of HGPRT-I signal and a significant decrease in HGXPRT expression (Fig 5C, bottom panel).

Since the 10% FBS present in regular HMI-9 medium most likely contains diminutive amounts of additional purines (i.e. adenine and adenosine) [33] which could have supported the observed reduced growth of the DKD HGPRT-I/HGXPRT cell line, we tested all generated RNAi cell lines in media containing dialyzed FBS serum and only one defined source of the purine bases, i.e. hypoxanthine (50 μM, HMI-9^hypoxanthine^) or xanthine (50 μM, HMI-9^xanthine^).

**Fig 6:**
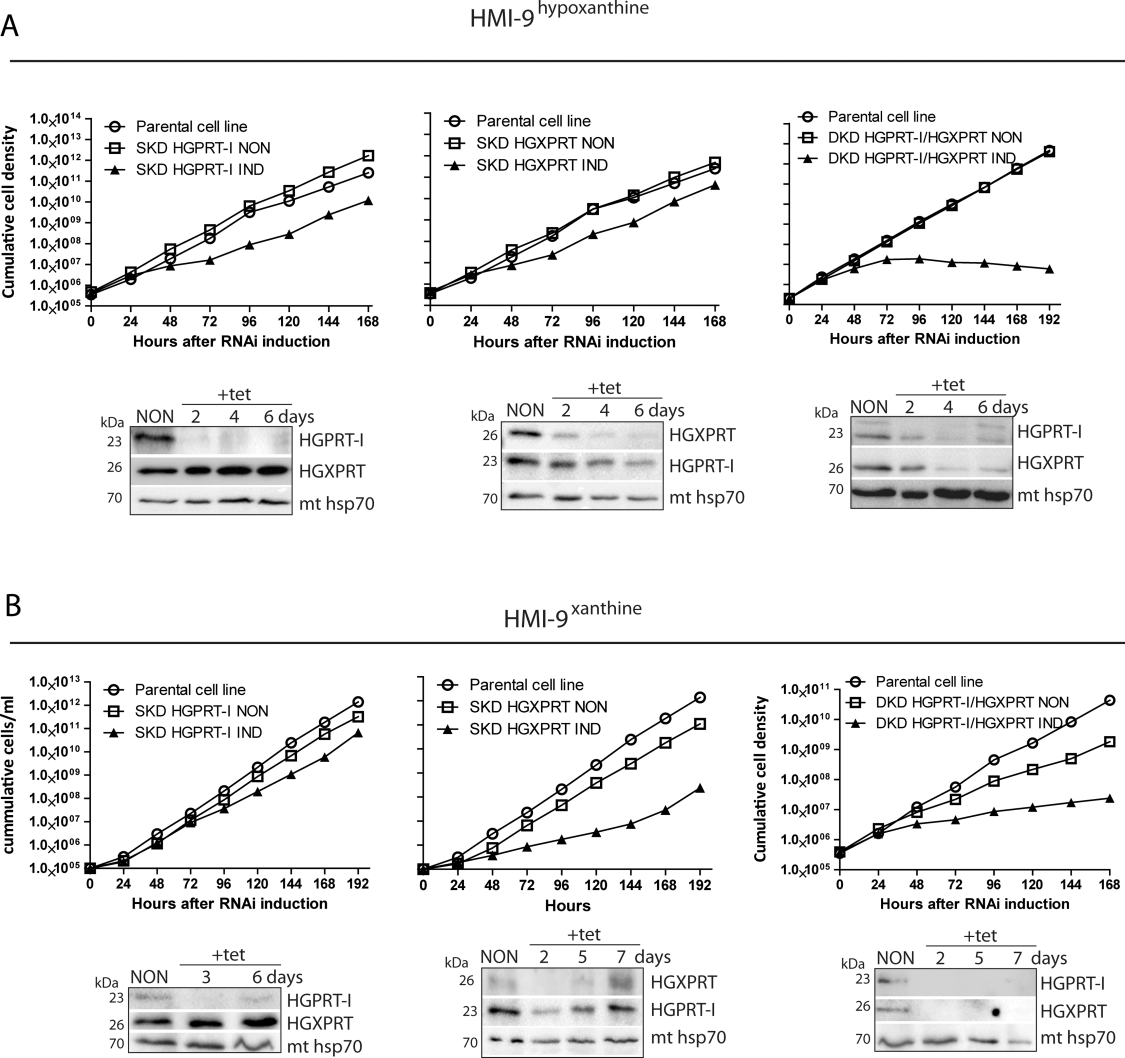
Effects of RNAi silencing of HGPRT-I and HGXPRT on *T*. *brucei* BF cell growth in the presence of indicated purine base. The RNAi cell lines were grown in HMI-9 medium containing hypoxanthine (A) or xanthine (B) as the sole purine source. The cumulative cell number was calculated from cell densities adjusted by the dilution factor needed to have the cultures at an appropriate concentration on each day. The steady-state abundance of HGPRT-I and HGXPRT in noninduced (NON) cells and in cells induced for given time points (bottom panels) was determined by immunoblotting using specific anti-HGPRT-I and anti-HGXPRT serums. Mt hsp70 served as a loading control. The protein marker sizes are indicated on the left.

While the noninduced cells grew normally with either of the added purines, the SKD HGPRT-I and SKD HGXPRT exhibited mild growth phenotypes in HMI-9^hypoxanthine^ confirming that either enzyme can utilize hypoxanthine (Fig 6A, left and middle panels). The slightly more profound growth phenotype in SKD HGPRT-I cells can be explained by the higher K_m_ value of HGXPRT for hypoxanthine compared to the K_m_ of HGPRT-I (13.7 *vs* 30.6 μM, Table 1). Western analysis of whole cell lysates of noninduced and RNAi induced cells confirmed effective and specific silencing of HGPRT-I and HGPXRT in the respective SKD cell lines (Fig 6A, left and middle panels). Densitometric analysis of protein bands did not reveal any significant changes in the steady state abundance of HGPRT-I and HGXPRT in the SKD HGXPRT and SKD HGPRT-I cell lines, respectively. Importantly, the DKD HGPRT-I/HGXPRT manifested significant growth phenotype in medium containing 50 μM hypoxanthine. Using the HGPRT-I and HGXPRT antiserums, a significant reduction in the expression of the targeted proteins was detected (Fig 6A, right panels). Our data confirms that there are no other enzymes that are able to convert hypoxanthine to IMP, a nucleoside monophosphate that can be further converted to XMP and other nucleoside monophosphates (Fig 1). Since xanthine becomes the dominant purine base to be salvaged when the parasite reaches the spinal fluids [46] we also tested the generated RNAi cell lines in the medium containing only this molecule (HMI-9^xanthine^). Surprisingly, xanthine is the least preferred purine for *in vitro* grown BF cells and the cells had to be cultured in this medium for two weeks to obtain regular growth. The noninduced and RNAi induced SKD HGPRT-I grew without any significant growth delays while the RNAi induced HGXPRT cells displayed a significant growth phenotype at day three upon the silencing (Fig 6B, left and middle panels). This result is in with agreement with the *in vitro* data showing that HGPRT-I has no affinity for xanthine. Thus, the cells expressing only HGPRT-I are not able to survive in medium exclusively containing this purine. Western blot analysis of the SKD HGXPRT cell line shows that while there was a significant down-regulation of HGXPRT expression two days after the tetracycline addition, at day seven we detected its noticeable re-expression. This observation suggests that some RNAi-induced cells bypassed the tetracycline repression, a phenomenon that has been commonly observed in *T*. *brucei* regulated expression systems [47]. The DKD HGPRT-I/HGXPRT cells grew poorly in the HMI-9^xanthine^ displaying a growth phenotype at day 2 after the RNAi silencing. The effective down-regulation of both enzymes was confirmed by Western blot (Fig 6B, right panels). These results highlight the importance of HGXPRT for the infectious stage of the parasites especially in the late phase of infection when the parasite crosses the blood brain barrier and xanthine becomes the main purine source.

### HGPRT-I, HGPRT-II and HGXPRT form homodimers *in vitro* and *in vivo*

**Fig 7:**
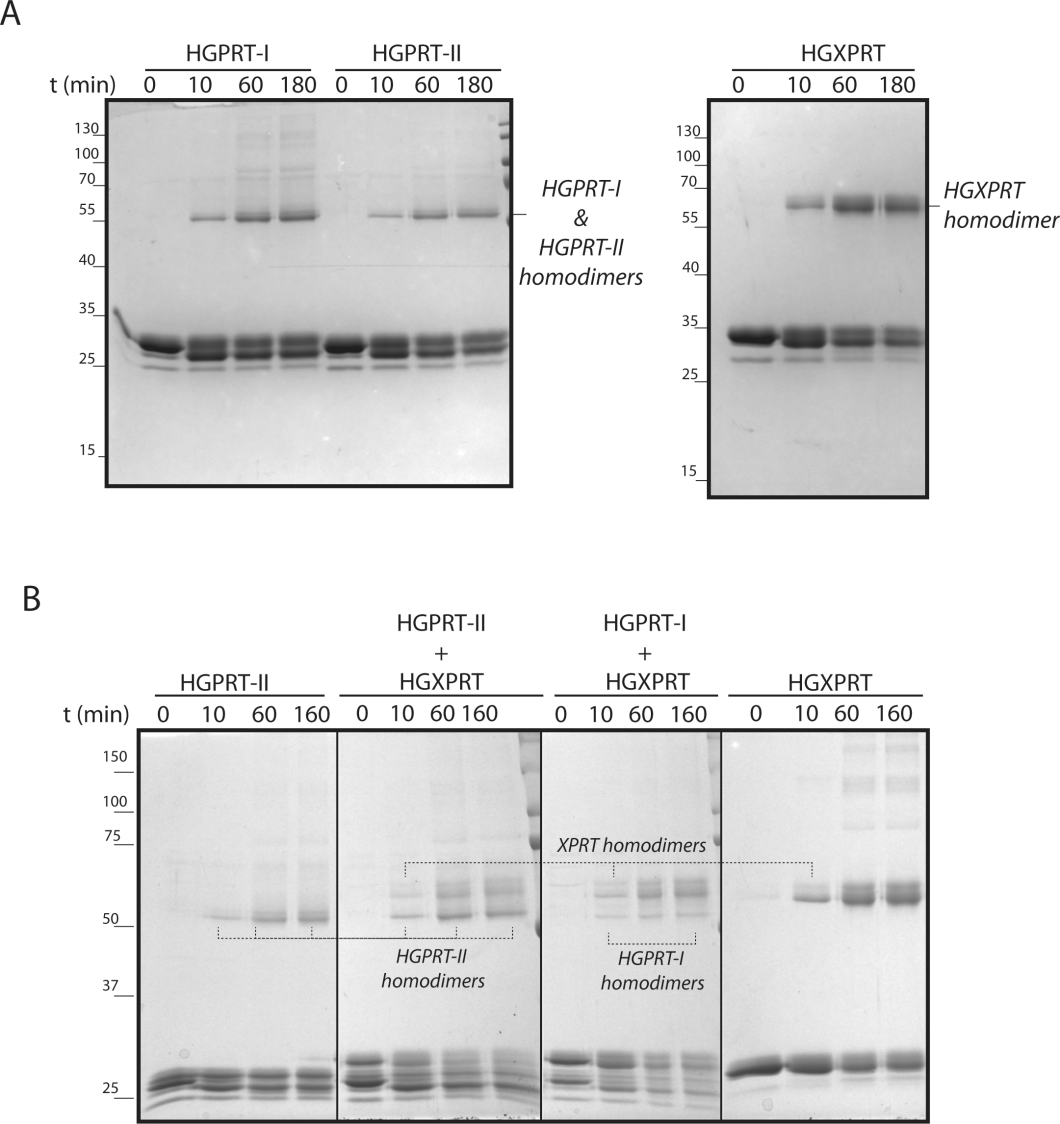
DMS crosslinking of recombinant HGPRT-I, HGPRT-II and HGXPRT. Proteins were crosslinked by DMS for 0, 10, 60 and 180 minutes and separated on the 15% SDS-PAGE followed by Coomassie R-250 staining. The HGPRT-I, HGPRT-II and HGXPRT homodimers were identified based on their sizes. The protein marker sizes are indicated on the left. (**A)** test for homodimerization, (**B)** test for heterodimerization.

The 6-oxopurine PRTases function as either dimers or tetramers in various protists, e.g. *Leishmania*, *Plasmodium* and *Toxoplasma* [48–50]. To establish the oligomeric properties of *T*. *brucei* HGPRT-I, -II and HGXPRT we used the purified recombinant proteins and performed a cross-linking experiment using dimethyl suberimidate (DMS). The cross-linked proteins were visualized by Coomassie staining on SDS-PAGE. Fig 7A shows that all three individual proteins are able to form homodimers. This result has subsequently been confirmed by analysis of the crystal structures of the two enzymes (see below), both of which reveal dimeric associations. When HGPRT-I or HGPRT-II were mixed together with HGXPRT no heterodimerization was observed as deduced from the positions of individual proteins on SDS PAGE gels (Fig 7B).

**Fig 8:**
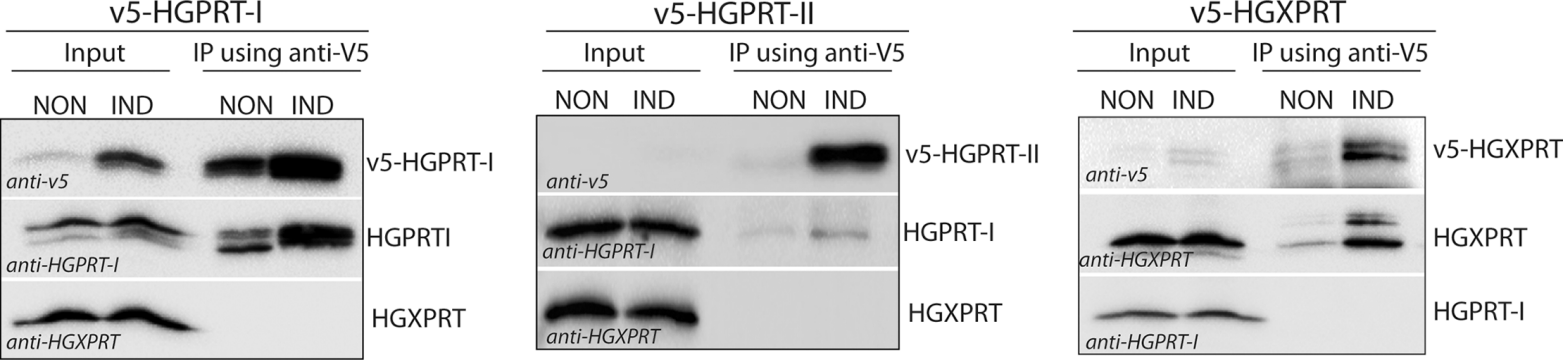
Immunoprecipitation of v5-tagged HGPRT-I, HGPRT-II and HGXPRT. Whole cell lysates of non-induced (NON) cells and cells induced for expression of v5-tagged HGPRT-I, HGPRT-II and HGXPRT (IND) were subjected to immunoprecipitation using anti-v5 monoclonal antibody. Immunoblots containing 5% input and 100% eluate were probed for the presence of v5-tagged HGPRT-I, HGPRT-II and HGXPRT, and for native untagged HGPRT-I and HGXPRT.

These results were confirmed *in vivo* using BF *T*. *brucei* cells overexpressing the v5-tagged versions of individual enzymes. Fig 8 shows that immunoprecipitation of v5-tagged HGPRT-I recovered the tagged and endogenous cytosolic HGPRT-I (Fig 8, left panel), while the immunoprecipitation of the v5-tagged glycosomal HGPRT-II pulled down only a negligible amount of the cytosolic HGPRT-I (Fig 8, middle panel). Therefore, it is possible that heterodimerization may happen between the HGPRT-I and HGPRT-II isoforms, but given the fact that expression of HGPRT-II is significantly reduced in the BF cells, and the HGPRT-I and HGPRT-II enzymes are confined to two different cellular compartments (i.e. cytosol and glycosome) it seems unlikely that these two proteins would form heterodimers *in vivo*.

Immunoprecipitation of v5-tagged HGXPRT recovered the tagged and endogenous HGXPRT, but not the HGPRT-I (Fig 8, right panel). We can therefore conclude that heterodimerization between HGPRT-I and HGPXRT does not occur either *in vivo* or *in vitro*.

### ANPs possess cytotoxicity effect on the BF *T*. *brucei* cells

**Fig 9:**
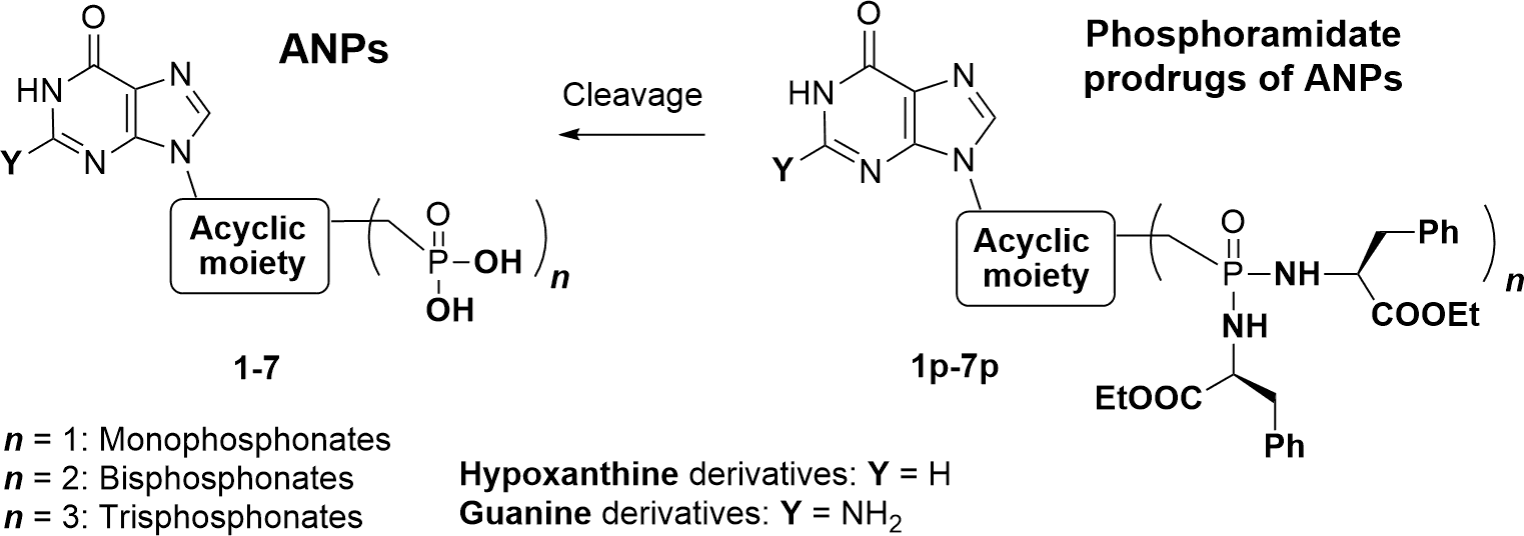
General structures of ANPs 1–7 and their phosphoramidate prodrugs 1p-7p.

**Fig 10:**
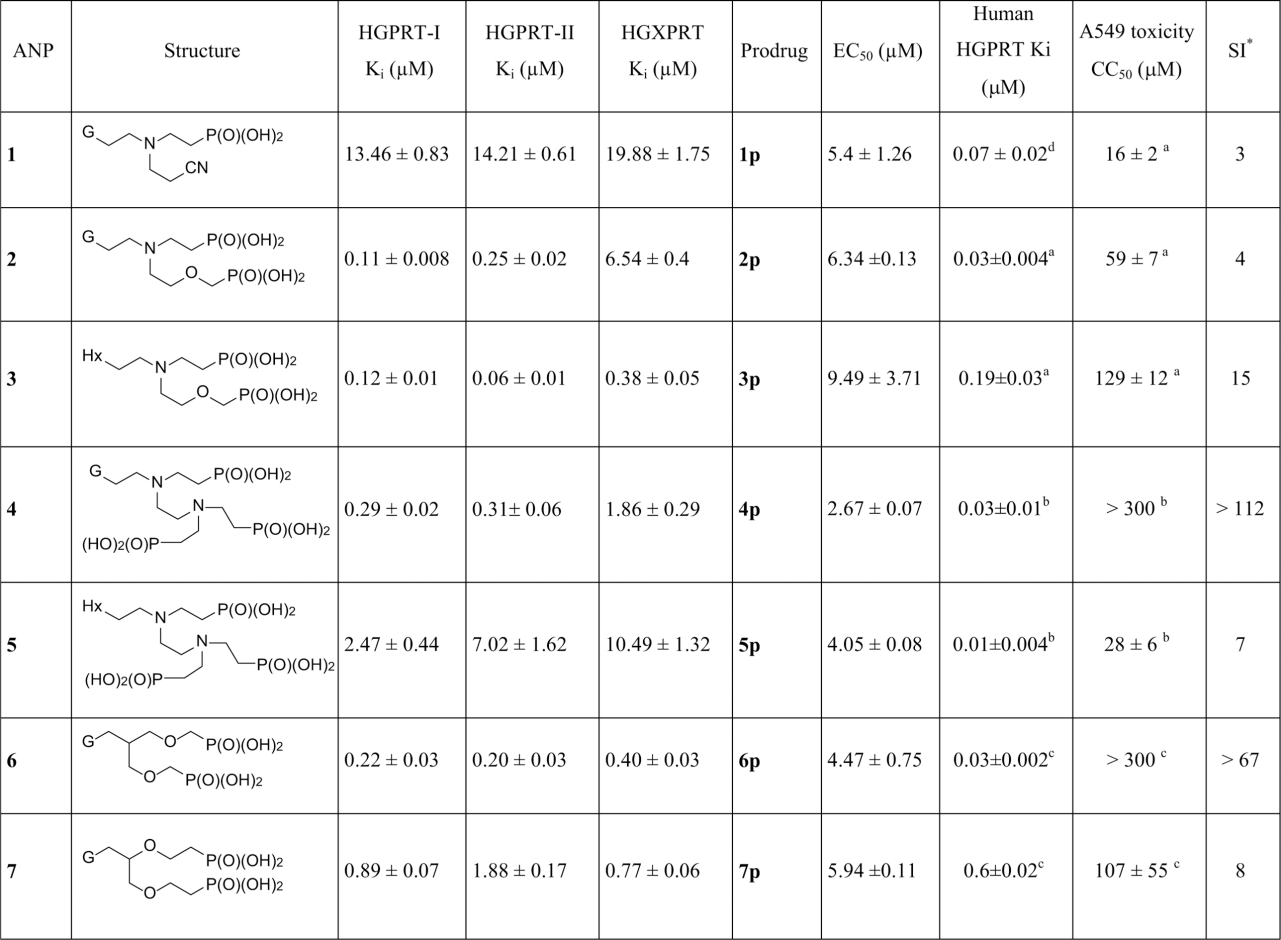
K_i_ values of the selected ANPs for HGPRT-I, HGPRT-II and HGXPRT and a comparison of the *in vitro* antitrypanosomal activity of their prodrugs against *T*. *brucei* BF427 cell line with their cytotoxicity in human cell lines. *Estimated selectivity index (SI) = average CC_50_ for A549 (Human lung carcinoma cells) divided by the average EC_50_ for *T*. *brucei* BF427 cell line. Synthesis of ANP inhibitors and their prodrugs is described in [20, 22, 28 and 29].^a^Data from [29]; ^b^Data from [28]; ^c^Data from [20]; ^d^Data from [22].

Over 100 acyclic nucleoside phosphonates were screened for their cytotoxic activity in BF *T*. *brucei* cells. Considering the polar character of the phosphonate group, the ANPs (examples 1–7) were tested mostly in the form of their cleavable prodrugs (examples 1p – 7p) to facilitate the transport across cell membranes (Fig 9, Fig 10) [30]. The most potent prodrug is compound 4p with an EC_50_ of 2.6 μM and with a selectivity ratio of >112, when compared with cytotoxic concentrations (CC_50_) in human cell lines [21,29,30]. The ANPs 1–7 corresponding to phosphoramidate prodrugs 1p-7p with the lowest EC_50_ values were further tested for their inhibition of HGPRT-I, HGPRT-II and HGXPRT. Both guanine- and hypoxanthine-based ANPs inhibited all three enzymes with K_i_ values ranging from 0.06 μM to 19.88 μM. The lowest K_i_ value (0.06 μM) was measured for compound 3 with HGPRT-II. There is a lack in correlation between the K_i_ values for the purified enzymes and the EC50 values for the prodrug (Fig 10). This could be partially explained by differences in the pharmacokinetics of the prodrug, i.e. the ability to enter the cells and to be cleaved. These attributes can significantly influence the correlation between the enzyme inhibition and prodrug activity. Nevertheless, the data illustrate that regardless of the purine base, individual ANPs can inhibit all three isoforms. This result may explain the observed cytotoxic effect of the tested ANPs in the culture as these compounds inhibit all three 6-oxopurine PRTases.

### Crystal structures of HGPRT-I and HGXPRT in complex with the ANP inhibitors

**Fig 11:**
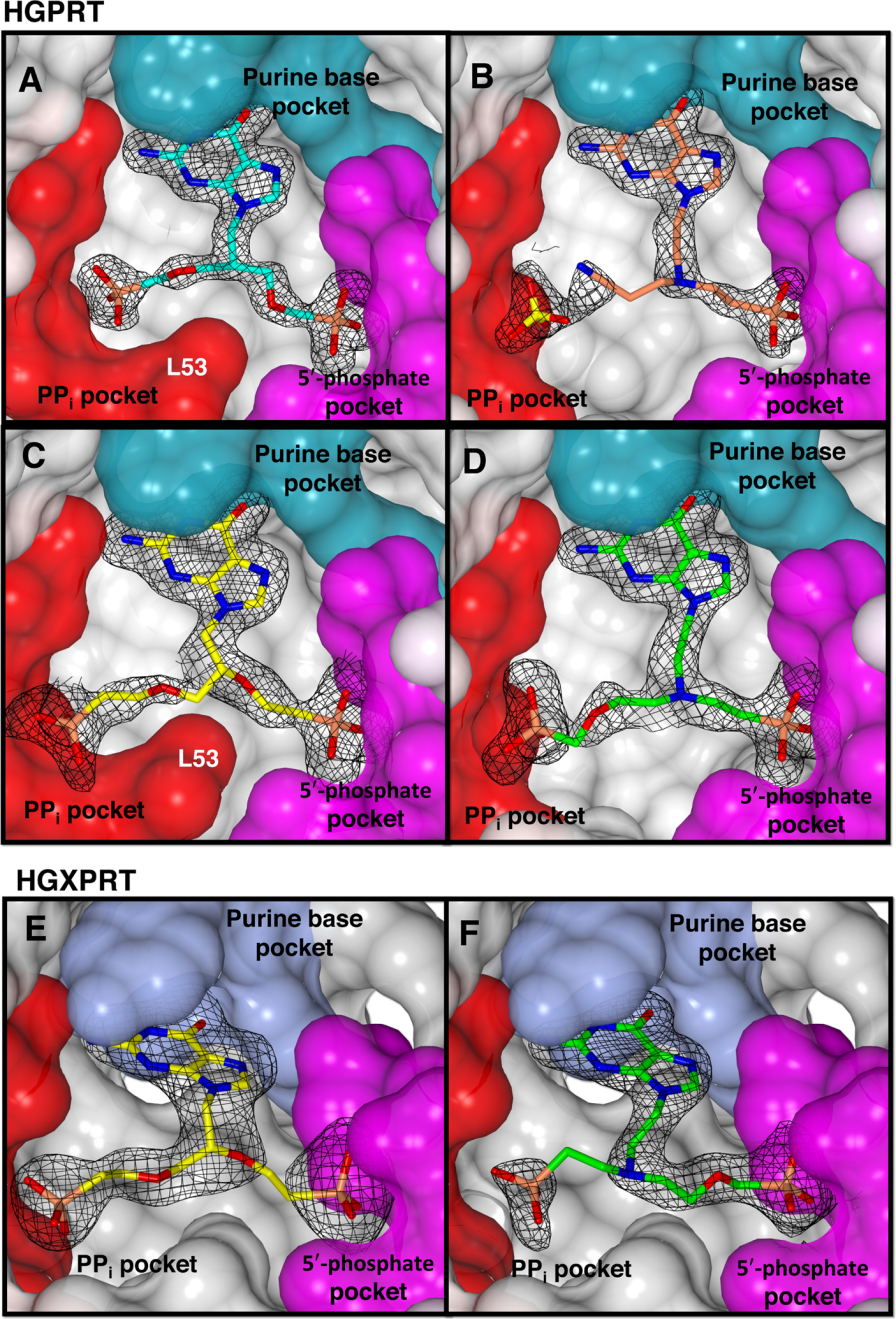
The active site of HGPRT-I and HGXPRT. Each enzyme is represented by its Connolly surface and the inhibitor is shown as a stick model. The F_o_-F_c_ electron density for each inhibitor is overlaid. (A) HGPRT-I.6 complex, (B) HGPRT-I.1 complex, (C) HGPRT-I.7 complex, (D) HGPRT-I.2 complex, (E) HGXPRT.7 complex and (F) HGXPRT.2 complex.

To explain the mode of binding of the ANPs, crystal structures of HGPRT-I in complex with compounds 1, 2, 6 and 7, and HGXPRT in complex with compounds 2 and 7 were determined. Given the conservation of active site residues in HGPRT-I and HGPRT-II, the crystal structure of HGPRT-II was not determined. The structure of HGPRT-I was previously solved in complex with five single chain ANPs and with nucleotide products GMP and IMP [16]. However, the structure of HGXPRT has not previously been determined. We defer a detailed analysis of the overall structure of this enzyme to a subsequent publication. Here, we describe the active site of this enzyme and its interactions with the ANPs. The structures were determined at resolutions that range from 1.76–1.99 Å for HGPRT-I and 2.26–2.64 Å for HGXPRT (Table 2). In both enzymes, the active site is situated at the interface of two domains referred to as the hood and core [16] and is primarily comprised of three pockets identified as the purine base binding pocket, the 5′-phosphate binding pocket and the pyrophosphate (PP_i_) binding pocket. Fig 11 shows the F_o_-F_c_ electron density and binding of 6 and 1 to HGPRT-I (Fig 11A and 11B) and the binding of 7 and 2 to both HGPRT-I and HGXPRT (Fig 11C, 11D, 11E and 11F).

**Table 2:** Data collection and refinement statistics for the HGPRT-I.ANPs and HGXPRT.ANPs complexes.

	HGPRT-I				HGXPRT	
Complex	compound 6	compound 7	compound 1	compound 2	compound 7	compound 2
***Crystal parameters***						
***a*, *b*, *c* (Å)**	116.0, 93.9, 44.7	44.7, 94.4, 110.0	94.9, 108.9, 45.0	89.5, 94.6, 109.7	61.3, 107.8, 117.7	61.3, 107.3, 117.4
***α*, *β*, *γ*(°)**	90, 107.8, 90	90, 90, 90	90, 90, 90	90, 90, 90	90, 96.3, 90	90, 96.61, 90
**Space group**	*C* 2_1_	*P* 2 2 2_1_	*P* 2 2 2_1_	*P* 2 2 2_1_	*P* 2_1_	*P* 2_1_
**Crystal size (mm)**	0.3 x 0.1 x 0.05	0.3 x 0.3 x 0.1	0.3 x 0.3 x 0.1	0.3 x 0.1 x 0.05	0.2 x 0.1 x 0.05	0.2 x 0.1 x 0.05
***Diffraction data***						
**Resolution range (Å)**	46.94–1.76	47.53–1.80	47.44–1.84	47.44–1.99	48.97–2.64	48.75–2.28
**Observations**	163324 (7357)	460142 (22439)	290920 (15619)	606011 (35194)	155191 (14848)	524870 (33272)
**Unique reflections**	44556 (2210)	43878 (2272)	41021 (2386)	64145 (3961)	44654 (4395)	68796 (4561)
**Completeness (%)**	98.9 (85.8)	98.8 (88.0)	99.6 (96.0)	99.0 (87.6)	99.2 (94.0)	99.9 (98.6)
***R***_***merge***_	0.142 (0.88)	0.133 (0.929)	0.160 (0.82)	0.181 (0.688)	0.076 (0.651)	0.102 (0.726)
***R***_***p*.*i*.*m*.**_	0.08 (0.561)	0.045 (0.316)	0.066 (0.371)	0.061 (0.240)	0.048 (0.521)	0.043 (0.309)
**<I>/<σ(I)>**	8.2 (1.5)	11.3 (2.6)	12.4 (2.4)	12.3 (3.7)	8.8 (1.3)	12.4 (2.0)
***Refinement***						
**Resolution limits (Å)**	1.760	1.79	1.84	1.99	2.638	2.280
***R***_**work**_	0.158	0.183	0.151	0.163	0.191	0.174
***R***_**free**_	0.192	0.203	0.189	0.198	0.249	0.216
**RMSD bond lengths (Å)**	0.018	0.012	0.016	0.009	0.003	0.004
**RMSD angles (**^**o**^**)**	1.561	1.223	1.389	0.998	0.594	0.604
Clashscore*	6.02	6.25	5.31	5.63	8.23	4.54
***Components of the asymmetric unit***						
**Dimers**	1	1	1	3	3	3
**Visible amino acids**	A: 2–80,101–204 B: 5–80,103–205	A: 4–80,103–204 B: 4–80, 103–198	A: 5–84,101–199 B: 4–81,102–202	A: 5–84, 101–200 B: 5–84, 103–205 C: 5–81, 103–199 D: 5–81, 103–201	A: 9-114/ 130–234 B: 8-114/ 130–234 C: 8-114/ 132–234 D: 8-114/ 133–234 E: 8-114/ 132–234 F: 8-114/ 131–234	A: 8-115/ 129–234 B: 8-114/ 130–234 C: 9-114/ 132–234 D: 9-114/ 132–234 E: 9-115/ 128–234 F: 8-114/ 129–234
**Ligands**	**PP352 (6)** x 2, SO_4_ x 1, PEG x 2, Tris x 2	**PP365 (7)** x 2, PEG x 2	**DA-XII-6 (1)** x 2, SO_4_ x 2, PEG x 2	**DA-XIII-44 (2)** x 4, PEG x 4	**PP365 (7)** x 6	**DA-XIII-44 (2)** x 6
**Waters**	418	287	467	684	53	658
**Mg**^**2+**^	2	0	2	8	2	8
***Ramachandran plot (%)***						
**Favoured**	97.2	96.8	97.1	96.9	96.48	97.8
**Outliers**	0	0	0.29	0.29	0.16	0.00

Values in parentheses are for the outer resolution shell.

$R_{merge}=\sum_{hkl}\sum_{i}|I_{i}(hkl)-(I(hkl))|/\sum_{hkl}\sum_{i}I_{i}(hkl)$

$R_{p.i.m.}=\sum_{hkl}{{\left[\frac{1}{\left[N(hkl)-1\right]}\right]}^{1/2}\sum }_{i}|I_{i}(hkl)-(I(hkl))|/\sum_{hkl}\sum_{i}I_{i}(hkl)$

where *I*_*i*_*(hkl)* is the observed intensity and *I*(*hkl*) is the average intensity obtained from multiple observations of symmetry related reflections. *Clashscores defined as the number of bad overlaps ≥ 0.4 Å per thousand atoms.

### Interactions in the purine base binding pocket

**Fig 12:**
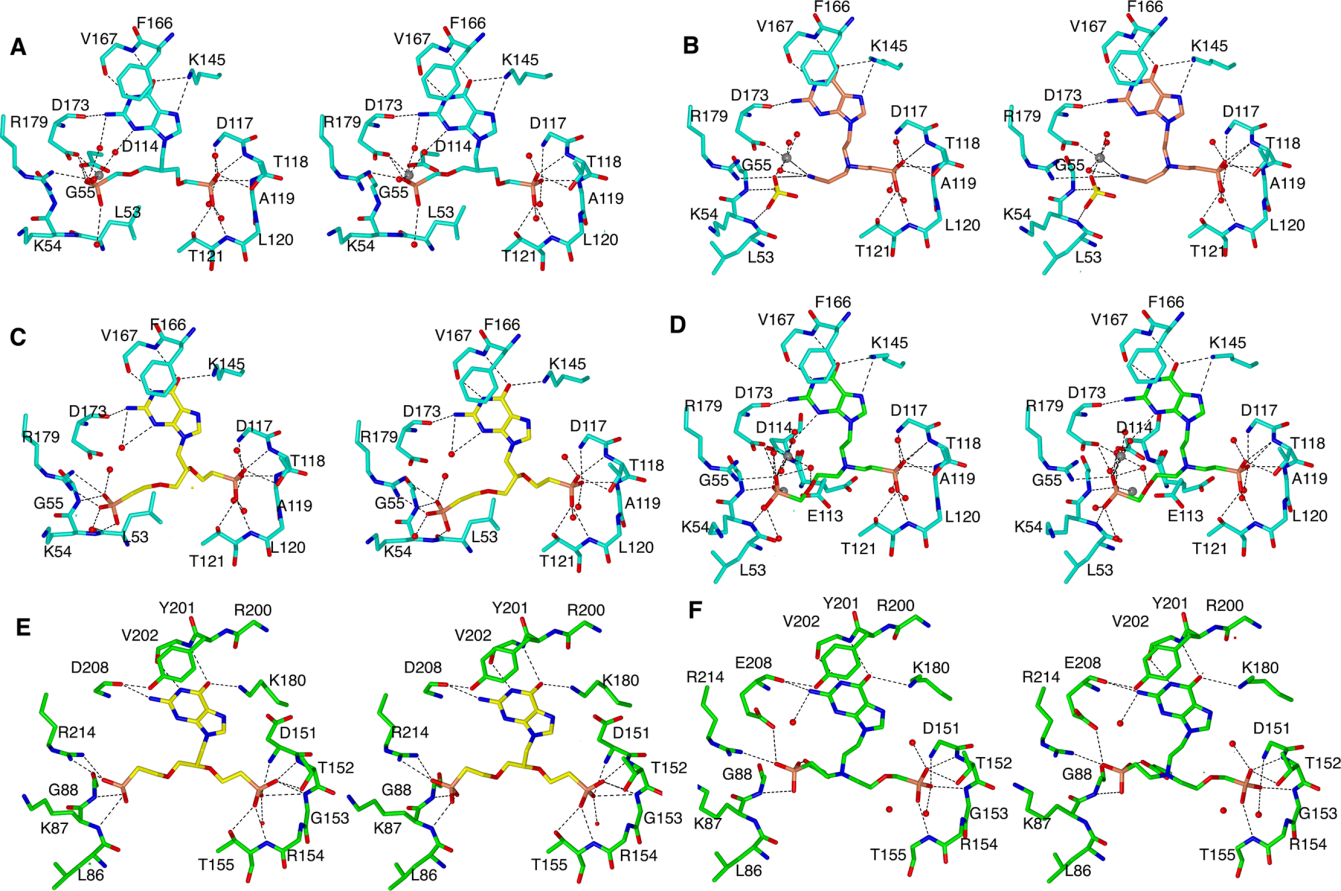
Stereoimages of the active sites of HGPRT-I and HGXPRT in complex with the four ANPs. **(A)** HGPRT-I. 6 complex (cyan carbon atoms), (**B)** HGPRT-I.1 complex (orange carbon atoms), (**C**) HGPRT-I.7 complex, (**D**) HGPRT-I.2, (**E**) HGXPRT.7 and (**F**) HGXPRT.2.

The guanine bases in 1, 2, 6 and 7, bind in a similar fashion to HGPRT-I and HGXPRT, being held in place by π-π stacking interactions with F166/Y201 (HGPRT-I/HGXPRT) (Fig 12) and forming at least four and up to six hydrogen bonds with the amino acid residues and a neighbouring water molecule. These hydrogen bonds are formed between (i) the NZ of K145/K180 and the 6-oxo group, (ii) the NZ of K145/K180 and N7 of the purine base in four of the complexes, (iii) the amide nitrogen of V167/V202 and N1 of the purine base (iv) the main chain oxygen of D173/D208 and the 2-amino group of the purine base, (v) the side chain of D208 to N2 and (vi) a water molecule and N3 of the purine base, in two of the complexes (Fig 12). Thus, there are few differences in how this base binds to both enzymes. This is a reflection of the fact that the K_m_ values for guanine are similar for these two enzymes (Table 1; 16 *vs* 32 μM).

### Interactions in the 5´-phosphate binding site

In all six complexes, a phosphonate moiety is located in the 5´-phosphate pocket (Fig 12). In HGPRT-I and HGXPRT, this pocket is formed by D117 to T121 and D151 to T155, respectively. In both complexes the backbone amide nitrogen atoms in this pocket hydrogen bond with the phosphoryl oxygen atoms of the ANP. Additional hydrogen bonds to the phosphoryl oxygen atoms also form with the side-chains of T118 and T121 in HGPRT-I and their counterpart residues, T152 and T155, in HGXPRT. In addition, in HGPRT-I there are two conserved water molecules that also hydrogen bond to the phosphoryl oxygen atoms. However, in the HGXPRT complexes no such water molecules are observed. We note here though, that the resolution of these structures is slightly lower than that for HGPRT-I. Therefore, the location of all the nearby waters may not be fully apparent in HGXPRT.

For compounds 2 and 7, we have determined structures of complexes with both HGPRT-I and HGXPRT, thus allowing a direct comparison of the binding modes of these two ANPs. As indicated above, a phosphonate group is observed in the 5´-phosphate pocket in all four structures (Fig 12B, 12C and 12D). When compound 7 binds, the phosphonate attached to the linker binds into the 5´-phosphate pocket in both complexes. However, compound 2 binds differently to the two enzymes. In HGPRT-I the phosphonate group attached to the linker binds into the 5´-phosphate pocket and the phosphonate in the attachment binds into the PP_i_ pocket (Fig 12D), whereas in HGXPRT the situation is reversed, with the phosphonate groups occupying alternate positions (Fig 12F).

### The pyrophosphate binding pocket

This is the location where most of the differences are observed when the binding modes of the six ANPs are compared (Fig 12). In the HGPRT-I.**6** complex the PP_i_ binding site is occupied by the phosphonate connected to the attachment. This phosphonate interacts with the side chain atoms of D114, D173, R179 and two water molecules (Fig 12A). In this complex the side chain of L53 points inwards towards the active site and the peptide bond between L53-K54 is in the *trans* conformation. This allows L53 to form hydrophobic interactions with three carbon atoms from the linker and attachments (Fig 12A). This *trans* peptide bond is also observed when HGPRT-I is in complex with ANPs containing a single phosphonate moiety with five or six carbons between the 6-oxopurine base and the phosphonate moiety [16]. However, in other crystal structures of HGPRT-I (e.g. in complex with GMP and IMP) the peptide bond between L53 and K54 can exist in the *cis* conformation, resulting in the side-chain of L53 being rotated out of the active site, allowing space for *P*Rib-*PP* to bind [16]. It would appear that in HGPRT-I the binding of *P*Rib-*PP* is the trigger for this conformational change, as has also been suggested to occur in other 6-oxopurine PRTases, including the human and *T*. *cruzi* enzymes [48,51].

In the HGPRT-I.1 complex, the PP_i_ binding site is occupied by a Mg^2+^ ion which ligands with the side chain of D173, three water molecules, and with the cyano moiety of compound 1 (Fig 12B). A sulphate from the crystallization buffer is bound in the PP_i_ binding site. This sulphate ion interacts with side chain of R179, and the amide nitrogen atoms of K54 and G55. In this complex the peptide bond between L53 and K54 is in the *cis* conformation (Fig 12B).

The phosphonate group of the attachment of compound 7 makes similar hydrogen bonding interactions with both enzymes (i.e. to the side chain atoms of D173/D208, R179/R214, and the amide nitrogen between L53/L86-K54/K87 and between G55/G88-K54/K87 (Fig 12C and 12E). However, in the HGPRT-I.**7** complex the peptide bond between L53-K54 is in the *trans* conformation and the side chain of L53 is inside the active site (Fig 12C). This allows L53 to form hydrophobic interactions with three carbon atoms in the linker and the attachment. In HGXPRT the peptide bond between L86-K87 is in the *cis* conformation, with the side chain of the L53 rotated out of the active site (Fig 12E).

In the HGPRT-I.2 complex the phosphonate attach to the linker occupies the 5´-phosphate pocket and the phosphonate connected to the attachment occupies the PP_i_ pocket (Fig 12D). However, in the HGXPRT.2 complex the situation is reversed and the phosphonate attached to the linker occupies the PP_i_ pocket and the phosphonate of the attachment occupies the 5´-phosphate pocket (Fig 12F). Although the phosphonate groups are swapped in the two structures the hydrogen bonding networks in the 5´-phosphate pocket are virtually identical in the two complexes. Within the purine binding pocket, there are more hydrogen bonds in the HGXPRT complex compared to the HGPRT-I complex. Of additional note is the fact that HGXPRT possesses a tyrosine at position 201, which forms a hydrogen bond with E208. In HGPRT-I this residue is phenylalanine and therefore no such bond can form. In the PP_i_ pocket, the phosphonate groups are oriented at slightly different angles in the two structures, this is due to the fact that the linkers/attachments are different and the presence of Mg^2+^ in the HGPRT-I complex. Nonetheless, both binding modes result in the L53-K54/L86-K87 peptide bond adopting a *cis* conformation. In this location, there are fewer interactions between the compound 2 and HGXPRT compared to HGPRT-I and this difference is the likely reason for this compound having a higher K_i_ value for HGXPRT compared to HGPRT-I (6.54 μM *vs* 0.11 μM). On the other hand, the binding mode of compound 6 in the two enzymes is very similar (Figs 12C and 11E) which is reflected in their almost identical K_i_ values (0.22 μM and 0.40 μM).

### Comparison of the binding modes of the ANPs with HGPRT-I, HGXPRT and human HGPRT ANPs

**Fig 13:**
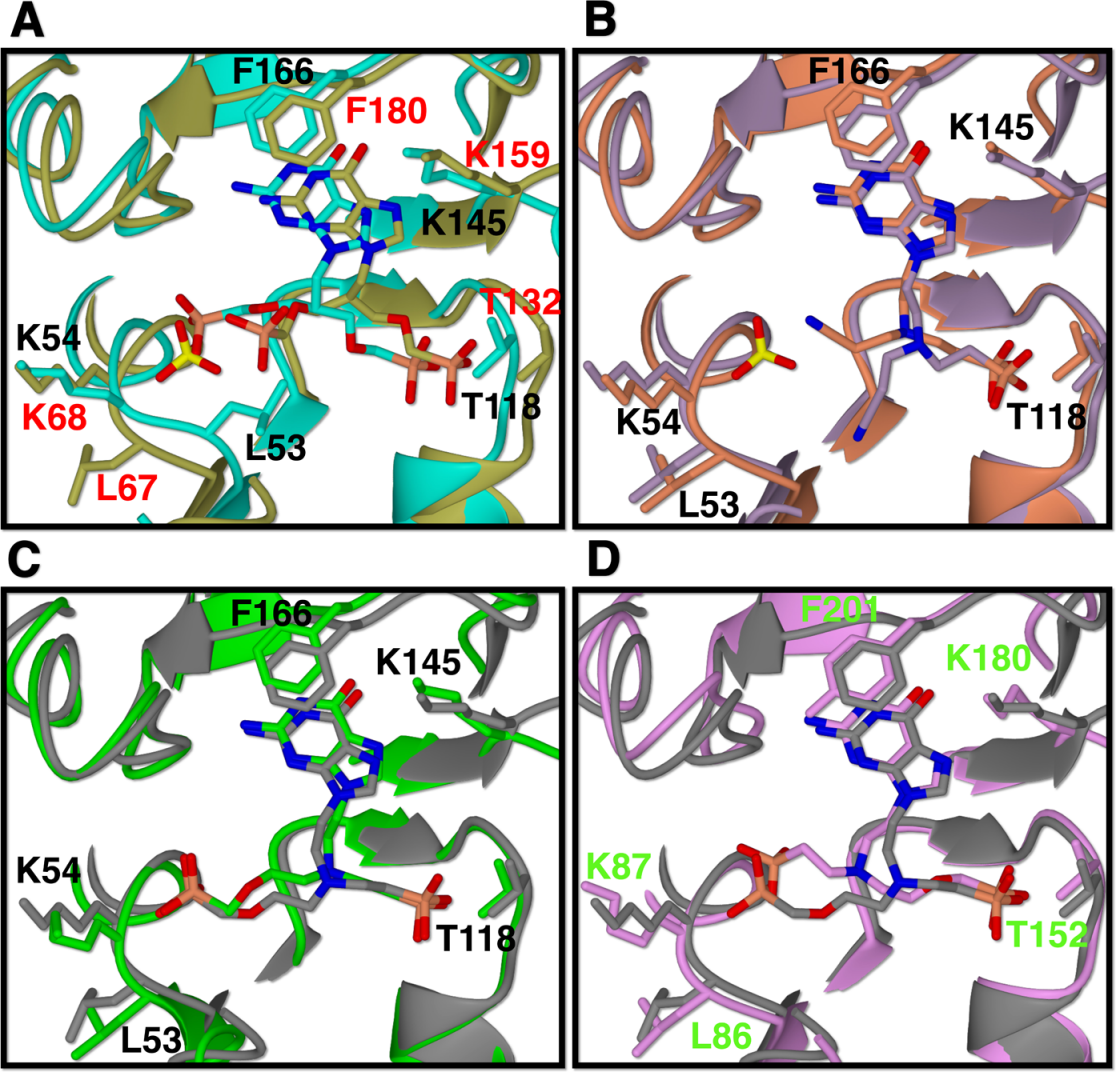
Active sites of HGPRT-I, HGXPRT and human HGPRT after superimposition. (A) compound 6 complexes. HGPRT-I. 6 in cyan, human HGPRT.6 in gold. (B) compound 1 complexes. HGPRT-I.1 in orange and human HGPRT.1 in purple. (C) compound 2 complexes. HGPRT-I.2 in green, human HGPRT.2 in grey (D) compound 2 complexes. HGXPRT.2 in pink, human HGPRT.2 in grey. Black labels are for HGPRT-I, green labels are for the HGXPRT and red labels are for human HGPRT.

The crystal structures of human HGPRT in complex with compounds 1, 2 and 6 have previously been determined (PDB codes 4IJQ, 4RAC and 4RAN). When the binding mode of compound 6 is compared with its binding mode to HGPRT-I there is a general displacement of the purine base, the linker and the attached phosphonate, as well as a different binding mode of the phosphonate joined to the attachment (Fig 13A). In addition, in HGPRT-I the L53-K54 peptide bond is in *trans* conformation and the side chain of L53 points into the active site, whereas in the human enzyme when compound 6 binds the equivalent peptide bond is in the *cis* conformation. Further, in the crystal structure of the human enzyme a sulphate ion fills the PP_i_ binding pocket, whereas there is no sulphate in the HGPRT-I and HGXPRT structures (Fig 13A). This difference appears to be a contributing factor in placing compound 6 in the two structures. By contrast, the binding of compounds 1 and 2 is very similar in HGPRT-I and human HGPRT (Fig 13B and 13C). However, the cyano group of the compound 1 does adopt a different orientation in the two enzymes. In HGPRT-I it is locked in place by the nearby magnesium ion, whereas in the human enzyme there is no such interaction.

Since the crystal structure of human HGPRT in complex with compound 2 is known this complex can be compared with HGXPRT. The main difference between the mode of binding of this compound in the two enzymes is that in HGXPRT the phosphonate group that belongs to the linker fills the PP_i_ pocket and the phosphonate group that belongs to the attachment is in the 5´-phosphate pocket, whereas in human HGPRT, the positions of the phosphonate groups of compound 2 are inverted (Fig 13D). Thus, there are several differences in the structures of human HGPRT and the trypanosomal enzymes that could be exploited for selective drug design.

## Discussion

The purine salvage pathway has been suggested as a possible drug target for a number of different parasites including *Plasmodium*, *Leishmania* and *Trypanosoma* species [52,53]. This is based on the fact that the *de novo* pathway for the synthesis of the nucleoside monophosphates is not present in any of these organisms. However, because of the redundancy in this pathway it was thought that none of the individual enzymes would be essential for the parasites. Here, we showed that double RNAi silencing of *T*. *brucei* HGPRT/HGXPRT caused significant growth phenotype *in vitro*. Moreover, selected prodrugs of potent ANPs had the ability to block the growth of BF parasites grown in culture. Interestingly, related prodrugs of aza-acyclic nucleoside inhibitors exerted cytotoxic activity against *P*. *falciparum* parasites also via inhibition of HGXPRT [29,30]. Although *Plasmodium* parasites utilize adenosine and inosine in addition to hypoxanthine, the first two are quickly converted to hypoxanthine by sequential actions of adenosine deaminase and purine nucleoside phosphorylase. Thus the enzymatic action of HGXPRT is crucial to produce IMP as the only precursor to all required nucleoside phosphate products. The indispensability of the plasmodial HGXPRT was further corroborated by metabolic studies [54]. Interestingly, while the *Leishmania* purine salvage pathway exhibits higher level of complementarity and redundancy compared to *Trypanosoma* and *Plasmodium* [52], the simultaneous knock-out of *L*. *donovani* HGPRT and XPRT enzymes was also lethal for the parasite grown *in vitro* [55]. Furthermore, uptake and salvage of adenine and adenosine was not sufficient to sustain the purine nucleoside pool suggesting that the interconversion enzymes (i.e. ADSL, ADSS, or GMP synthase), could be actually indispensable for this parasite as well. Thus, regardless of initial views, the purine salvage enzymes are possible targets in these parasites.

In the mammalian host *T*. *brucei* lives extracellularly and exploits environments rich mainly in hypoxanthine, xanthine and inosine. Other purine bases and nucleosides (e.g. adenine and adenosine) are present in negligible concentrations [46]. Therefore, the parasite should be susceptible to inhibition of not only the salvage enzymes, like HGPRT and HGXPRT (shown here), but also to the key interconversion enzymes, such as GMPS. Indeed, GMPS knock-out cell line was not able to produce infection in mice and specific GMPS inhibitors arrested the parasite´s growth *in vitro* [53]. More importantly, RNAi silencing of ADSS and ADSL, enzymes responsible for interconversion of IMP to AMP, caused attenuated virulence of *T*. *brucei* in mice suggesting that in mouse plasma there are limited concentrations of adenine and adenosine [56]. As a result, it is plausible to expect that HGPRT/HGXPRT would be also essential in the animal model. Moreover, the obvious promiscuity of *T*. *brucei* HGXPRT offers an elegant solution of one drug to target both HGPRT and HGXPRT enzymes and thus pharmacological inhibition of both enzymes can be proposed.

Crystal structures of the two 6-oxopurine PRTs in complex with the ANPs (Table 2) provide the specific details of their binding modes. As it has been also observed in studies of *P*. *falciparum* HGXPRT [25], five atoms appears to be the optimal length of the linker to form interactions between a phosphonate end and residues in the 5´-phosphate pocket. The number of atoms required to be attached for the linker to reach into the PP_i_ pocket is of the order of 5 to 6 depending on the location of the attachment, the presence or absence of magnesium and whether or not the PP_i_ binding loop has a *cis* peptide bond present. The kinetic data show that compounds that possess guanine as the base are effective at inhibiting all three isoforms of the 6-oxopurine PRTs, and this appears to translate into compounds that are effective in killing *T*. *brucei* parasites in culture with the prodrug versions of these compounds. Compounds 3 and 6 are the most broadly effective inhibitors tested so far with *K*_i_ values lower than 1 μM for all three enzymes. Another promising inhibitor is ANP 4 with a very favourable selectivity index. Compound 1 is also of interest for further development since its K_i_ value is quite high compared to the antitrypanosomal activity of its prodrug (relative to the other compounds). This is likely due to the fact that there is only one phosphonate group that is required to be masked by the prodrug formulation. The structure of HGPRT-I has a highly unusual PP_i_ binding site in that, that in the *trans* conformation leucine points inward toward the 5’-phosphate pocket. This is a feature that could be utilized to develop inhibitors that are highly specific for this enzyme. Moreover, the PPi binding pocket differs at amino acid position 54 between HGPRT-I and HGPRT-II isoforms. HGPRT-I contains a lysine at this position whereas HGPRT-II contains arginine. Based on previous crystal structures of the 6-oxopurine PRTases, movement of this side chain is critical for opening up the active site to allow access to *P*Rib-*PP*. It is possible that an arginine in this position could form more intramolecular interactions retarding the ability for *P*Rib-*PP* to rapidly bind to the enzyme and or for the enzyme to rapidly release the product [57]. In this context, a sequence comparison across the 6-oxopurine PRTases shows that lysine is the preferred residue at this location [21,28,49,51,57,58]. HGXPRT is markedly different from other 6-oxopurine PRTases in that it possesses the ability to use xanthine as a substrate. This is a difference compared to the human enzyme that could also be exploited for the development of specific inhibitors. Importantly, the need to develop highly selective inhibitors of the parasite enzyme does not apply here. Humans also possess the *de novo* pathway for synthesis of their DNA/RNA and thus they are not fully reliant on the salvage pathway. This is demonstrated by individuals in the community where mutations occur to the human HGPRT resulting in a loss of 90% of activity of this enzyme, yet individuals with this affliction experience few symptoms, all of which are readily treatable by application of allopurinol [59].

In conclusion, we have demonstrated that *T*. *brucei* parasites require activity of HGPRT and HGXPRT enzymes for the normal cell growth *in vitro*. ANPs represent a promising class of potent and selective compounds as they inhibit the enzymes with K_i_ values in nanomolar range and exert cytotoxic effects on *T*. *brucei* cells grown *in vitro* with EC_50_ values in the single digit μM range. Our results provides a foundation for further investigations of these compounds *in vivo* and suggests that HGPRT-I/HGPRT-II/HGXPRT could be possible targets for future drug discovery efforts directed at controlling HAT.

## Supporting information

S1 Supplementary File List of oligonucleotides used in this study. (DOCX)
