# Supplementary material for: Evaluation of the Trypanosoma brucei 6-oxopurine salvage pathway as a potential target for drug discovery
Source: PLoS Negl Trop Dis. 2018 Feb 26;12(2):e0006301. doi: 10.1371/journal.pntd.0006301 (PMC5843355; doi:10.1371/journal.pntd.0006301)
Supplement: S1 Supplementary File — (DOCX) [file pntd.0006301.s001.docx]

**Supplementary file S1.** List of oligonucleotides used in this study

HGPRT-I p2T7-177 RNAi forward primer: 5'- GACGGATCCACGACTTCGCAA-3'

HGPRT-I p2T7-177 RNAi reverse primer: 5'-GTGAAGCTTGTCGATAGCCACG-3'.

HGXPRT p2T7-177 RNAi forward primer: 5'- CACAAGCTTTGCACTCGGGCCATCCTCTCA-3'

HGXPRT p2T7-177 RNAi reverse primer: 5'-CACCTCGAGGTACGCCCCGTGTCGGCAAT-3'.

HGPRT-I pT7-V5 N-term forward primer: 5´-TACGGATCCGAACCAGCTTGCAAATAC-3'

HGPRT-I pT7-V5 N-term reverse primer: 5'-CACTCTAGATTACCGCTTGGCTTCTC-3'

HGPRT-II pT7-V5 N-term forward primer: 5'- TACGGATCCGAACCAGCTTGCAAATAC -3'

HGPRT-II pT7-V5 N-term reverse primer: 5'- GCGTCTAGATTACAGTTTTGCCTTCACAGC-3'

HGXPRT pT7-V5 N-term forward primer: 5'- CACGGATCCCACTCGGGCCATC-3'

HGXPRT pT7-V5 N-term reverse primer: 5'- CACTCTAGATTACAATTTGCTCG-3'

HGPRT-I pSK B3 overexpression forward primer: 5'-CACGGATCCGAACCAGCTTGCAAATACG -3'

HGPRT-I pSK B3 overexpression reverse primer: 5'-GTGCTCGAGTTACCGCTTGGCTTCTCC-3'

HGPRT-II pSK B3 overexpression forward primer: 5'- CACGGATCCGAACCAGCTTGCAAATACG-3'

HGPRT-II pSK B3 overexpression reverse primer: 5'- GTGCTCGAGTTACAGTTTTGCCTTCACAGCG-3'

HGXPRT pSK B3 overexpression forward primer: 5'-CACGGATCCCACTCGGGCCATCCTCTC-3'

HGXPRT pSK B3 overexpression reverse primer: 5'-GTGCTCGAGTTACAATTTGCTCGGGTACC -3'
